# Integrated Genomic and Transcriptomic Analyses Reveal a Two-Tier Adaptive Strategy for Wheat Root Salt Tolerance: Constitutive Auxin Biosynthetic Capacity and Stress-Responsive Transcriptional Repression

**DOI:** 10.3390/biology15120965

**Published:** 2026-06-19

**Authors:** Kyung-Hee Kim, Ji Yu Jeong, Taekyeom Kim, Sang Yong Park, Byung-Moo Lee, Jae Yoon Kim

**Affiliations:** 1 Department of Life Science, Dongguk University, Seoul 04620, Republic of Korea; redanan@dongguk.edu; 2 Department of Plant Resources, College of Industrial Science, Kongju National University, Yesan 32439, Republic of Korea; 02jjy04@gmail.com (J.Y.J.); 211479@kongju.ac.kr (T.K.); sangyongpark@kongju.ac.kr (S.Y.P.)

**Keywords:** *Triticum aestivum*, salt stress, root system architecture, genome-wide association study (GWAS), transcriptomics, auxin biosynthesis, *TaIAO*, transcriptional repression

## Abstract

Soil salinity is a major constraint on global wheat productivity, yet the genetic and molecular determinants of root system architecture (RSA) adaptation under salt stress remain poorly characterized. We integrated a genome-wide association study (GWAS) of 566 wheat accessions with comparative RNA-seq transcriptomics to identify the genetic and transcriptional determinants of RSA adaptation under 200 mM NaCl. GWAS identified a candidate locus on chromosome 7B harboring *TaIAO*, which encodes a protein with predicted aldehyde oxidase-like activity consistent with a role in tryptophan-dependent auxin biosynthesis. Accessions carrying the favorable CC allele exhibited significantly greater root volume retention than those carrying the GG genotype (*p* < 0.001). Comparative RNA-seq revealed that the salt-tolerant Sarajevo 1 exhibited coordinated transcriptional repression of three distinct modules—cell wall expansion (*TaExpansin*), auxin redistribution (*TaPIN-like*), and stress-associated ROS defense (*TaPOD1*)—whereas the sensitive genotype CI 17260 aberrantly induced or incompletely repressed these modules under stress. ELISA-based IAA quantification, ROS imaging, and qRT-PCR analysis provided independent physiological and transcriptional support for these patterns. These findings support a two-tier adaptive model in which constitutive genetic variation at the *TaIAO* locus may contribute to a developmental baseline, coupled with coordinated stress-responsive transcriptional repression of energy-consuming modules, providing promising targets for marker-assisted breeding of salt-tolerant wheat.

## Simple Summary

Soil salinity threatens over 20% of global wheat cultivation, severely impairing root development and reducing grain yield. However, the genetic basis underlying root salt tolerance remains poorly understood. By screening 566 wheat accessions, we identified a genomic region on chromosome 7B associated with auxin biosynthesis. Accessions carrying favorable alleles at this locus retained 95.2% of their root length under salt stress, compared to only 61.1% in sensitive lines. Our results suggest that salt-tolerant wheat may employ a two-tier adaptive strategy: inherent root developmental capacity associated with *TaIAO*, coupled with stress-responsive transcriptional repression of energy-consuming modules involved in cell wall expansion, auxin redistribution, and reactive oxygen species (ROS) defense. In contrast, the sensitive genotype aberrantly induced or incompletely repressed these modules under stress, resulting in more severe root architectural deterioration. These findings provide promising genetic targets for the marker-assisted breeding of salt-tolerant, climate-resilient wheat cultivars.

## 1. Introduction

Wheat (*Triticum aestivum* L.) is one of the most important staple crops globally, yet its productivity is increasingly threatened by soil salinity, which affects more than 20% of cultivated land worldwide and causes annual economic losses exceeding 27 billion USD [1,2]. Although wheat is considered moderately tolerant to salinity [2,3,4,5], grain yield losses exceeding 40–50% have been reported under moderate-to-severe stress conditions [6,7]. Salinity-induced impairment of root function—including disruption of ion homeostasis, nutrient acquisition, and root system architecture (RSA)—represents a primary constraint on productivity [8].

**Figure 1:**
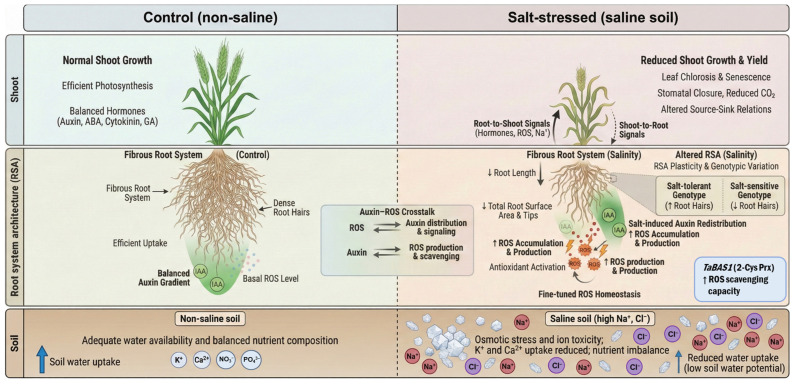
Schematic representation of soil–root–shoot interactions and regulatory mechanisms in wheat under salinity stress [5]. The diagram contrasts wheat plant responses under non-saline control (**left**) and salt-stressed (**right**) conditions. Key mechanisms: (i) soil ion dynamics: elevated Na^+^ and Cl^−^ concentrations impose osmotic stress and ion toxicity, restricting water and nutrient (K^+^, Ca^2+^) uptake [12]; (ii) RSA remodeling: root plasticity is coordinated through the selective regulation of auxin–ROS modules [13,14], including constitutive antioxidant systems such as *TaBAS1* (a 2-Cys peroxiredoxin), whose stress-responsive transcriptional regulation contributes to metabolic economy under salt stress [15]; and (iii) shoot responses: root-derived stress signals promote stomatal closure and reduce photosynthesis to limit oxidative damage [5,12].

Salinity perturbs water uptake and ion homeostasis, thereby inhibiting root growth, disrupting nutrient acquisition [9,10,11]. Salinity-induced alterations of RSA, including reductions in root length, surface area, and root hair development, have been recognized as critical but underexploited targets for enhancing wheat salt tolerance. To provide a holistic view of these multiscale responses, Figure 1 presents a schematic of the regulatory mechanisms operating along the soil–root–shoot continuum under salinity stress. The figure highlights how primary stressors at the soil level initiate a cascade of physiological and molecular responses [12], positioning RSA remodeling as a central regulatory hub driven by the crosstalk between auxin signaling and reactive oxygen species (ROS) homeostasis [13,14]. These root-level adjustments, modulated by antioxidant defenses such as *TaBAS1* (a 2-Cys peroxiredoxin that enhances ROS detoxification capacity [15]), subsequently feed back to the shoot, impairing whole-plant growth and development, and thereby constraining productivity under saline conditions.

RSA plasticity enables plants to dynamically reconfigure root growth to optimize soil exploration and resource capture under heterogeneous and stressful environments. Under salinity, many species display reduced primary root elongation but enhanced lateral root proliferation or root hair formation, reflecting a shift from vertical foraging to more localized resource acquisition [16]. In wheat, salinity decreases seminal root length, total root surface area, and root tip number, although considerable genotypic variation exists in the extent of these responses [17]. Understanding the genetic and molecular basis of such adaptive root strategies is therefore essential for breeding salt-tolerant cultivars.

Auxin is a central regulator of RSA plasticity, integrating environmental cues with developmental programs that control root elongation, branching, and gravitropic set-points [18]. Salt and osmotic stress modulate auxin biosynthesis, transport, and signaling to re-pattern root growth, often through differential auxin distribution between the root tip and differentiation zone. Salt-induced changes in auxin gradients have been linked to reduced root meristem activity but increased lateral root formation in model species, highlighting the importance of auxin homeostasis for stress-responsive RSA remodeling. Emerging evidence suggests that similar auxin-mediated mechanisms contribute to root architectural responses to salinity in cereals, although the underlying regulatory networks in wheat remain poorly characterized at the genome scale [18,19].

Beyond its roles in root elongation and lateral root initiation, auxin is an established regulator of root gravitropism—the directional growth response that orients roots toward gravity, which is essential for effective soil exploration. Upon gravitropic stimulation, amyloplasts in the columella cells of the root cap sediment toward the lower cell membrane, triggering lateral redistribution of auxin mediated by PIN3 and PIN7 efflux carriers [20,21]. This generates a transient auxin gradient between the lower and upper flanks of the root tip; the higher auxin concentration on the lower flank suppresses cell elongation, thereby inducing the downward curvature characteristic of positive gravitropism [20,21]. Under salinity stress, NaCl-induced changes in PIN carrier abundance and membrane polarity disrupt this gravitropic auxin gradient, impairing the capacity of the root to maintain its gravitropic set-point angle [22,23]. This perturbation of PIN-mediated gravitropic auxin distribution is consistent with the broader dysregulation of auxin transport reported in salt-sensitive root responses, underscoring the importance of precise PIN-mediated auxin flux for adaptive root architecture and function under saline conditions.

A key but underexplored node in auxin homeostasis is the terminal biosynthetic step catalyzed by aldehyde oxidases (AO; EC 1.2.3.7), which convert indole-3-acetaldehyde (IAAld) to bioactive indole-3-acetic acid (IAA) and constitute a conserved multigene family in higher plants [24]. In *Arabidopsis*, four AO isoforms (AtAAO1–AtAAO4) have been functionally characterized, with AtAAO1 demonstrating specific activity toward IAAld in the tryptamine branch of tryptophan-dependent auxin biosynthesis [24]. In wheat, AO-family genes have been identified on chromosomal groups 2, 5, and 7 and studied primarily in the context of abscisic acid (ABA) and carotenoid metabolism in grain [25]; however, whether natural variation in AO-mediated auxin biosynthetic capacity contributes to genotypic differences in root architectural plasticity under salinity has not been investigated. *TaIAO* (*Triticum aestivum* Indole-3-Acetaldehyde Oxidase-like; *TraesCS7B02G418900*) represents one such candidate AO-family gene in wheat, located on chromosome 7B, whose potential role in root auxin biosynthesis under salinity is addressed in this study.

ROS represent another key component of root adaptation to salt stress. Wheat roots exposed to NaCl exhibit altered ROS accumulation and activation of antioxidant enzymes such as peroxidases, catalases, and peroxiredoxins; manipulation of ROS-scavenging genes has been shown to enhance salt tolerance. For instance, overexpression of the wheat 2-Cys peroxiredoxin gene *TaBAS1* improves salt and oxidative stress tolerance in both wheat and *Arabidopsis* by strengthening ROS detoxification capacity [15]. These observations point to a crucial role of finely tuned ROS homeostasis in maintaining root growth and integrity under salinity [26]. Class III peroxidases catalyze oxidative cross-linking of cell wall polymers, contributing to both cell wall stiffening and apoplastic H_2_O_2_ consumption [27]; controlled expression of class III peroxidase genes has been shown to influence salt tolerance in wheat [28]. *Expansins* mediate cell wall loosening by disrupting non-covalent hydrogen bonds within the polysaccharide matrix, enabling turgor-driven cell elongation [29,30]; under salinity, the dosage and directionality of expansin gene expression have been shown to influence salt tolerance outcomes in wheat [30,31]. Recent studies have highlighted intimate crosstalk between auxin and ROS during root development, whereby ROS influence auxin distribution and signaling, and auxin, in turn, modulates ROS production and scavenging systems. However, the extent to which auxin signaling and ROS-related defense mechanisms are jointly deployed to orchestrate wheat RSA plasticity under salt stress, and how this coordination differs between tolerant and sensitive genotypes, remains incompletely understood, underscoring the need for integrative genomic and transcriptomic approaches in genetically diverse populations [32].

The Korean Wheat Core Collection (KWCC) is a genetically diverse panel derived from a large wheat germplasm set and has been extensively characterized for genetic diversity, population structure, and agronomic traits, providing a valuable resource for dissecting complex stress-response mechanisms [33,34]. Building on this resource, we hypothesized that natural variation in root plasticity under salt stress is governed not merely by the activation of classical stress-defense pathways, but potentially by a strategy of profound metabolic economy, hereafter referred to as metabolic quiescence. We propose that tolerant genotypes strategically repress energy-consuming transcriptional modules including those involved in auxin redistribution, cell wall expansion, and constitutive ROS scavenging, to preserve root architecture under salinity. In this study, we integrated GWAS of root morphological traits with comparative RNA-seq of contrasting wheat accessions to determine whether the mechanistic hallmark of salt-tolerant root adaptation in wheat resides in the coordinated transcriptional repression of auxin, expansin, and peroxidase modules, rather than in their stress-induced activation.

## 2. Materials and Methods

### 2.1. Plant Materials

A total of 566 wheat (*Triticum aestivum* L.) accessions from the Korean Wheat Core Collection (KWCC) were used in this study. The KWCC was originally established by the National Institute of Crop Science (NICS), Rural Development Administration (RDA), Korea, to represent the genetic diversity of wheat germplasm cultivated, collected, and conserved in Korea [33]. The population structure and genetic diversity of these accessions have been characterized in previous studies [33,34]. All 566 accessions were phenotyped for root morphological traits under control and 200 mM NaCl conditions, and mean trait values served as phenotypic inputs for GWAS (Section 2.4). Three genotypes were subsequently selected for in-depth analysis based on two criteria: (1) root traits performance under salt stress, whereby Accession 1936 (cv. Sarajevo 1) was classified as salt-tolerant and Accession 1694 (cv. CI 17260) as salt-sensitive; and (2) haplotype at the peak GWAS SNP (AX-95079518) on chromosome 7B, with Sarajevo 1 carrying the favorable CC haplotype and CI 17260 carrying the unfavorable GG haplotype. Accession 305 (cv. Keumkang), a locally adapted Korean cultivar, was included as a reference genotype.

### 2.2. Hydroponic Culture and Salt Treatment

Seeds were surface-sterilized with 2% (*v*/*v*) sodium hypochlorite solution for 10 min and rinsed thoroughly with distilled water. To promote uniform germination, seeds were imbibed in distilled water for 24 h at 4 °C in the dark. Seeds were then placed on moistened germination paper in Petri dishes and grown for 10 days. Tap water was used as the culture medium throughout, as preliminary experiments indicated superior seedling establishment compared with distilled water; background NaCl levels in municipal tap water are negligible relative to the 200 mM NaCl treatment concentration, and both control and salt-stressed plants were maintained under identical tap water conditions. Uniform seedlings were transplanted into sponge supports and placed in hydroponic trays (Supplementary Figure S1). Plants were grown in a controlled growth chamber under a 16 h light/8 h dark photoperiod, a day/night temperature of 23 °C/18 °C, and 60% relative humidity. For the salt stress treatment, seedlings were exposed to 200 mM NaCl dissolved in tap water; control plants were maintained in tap water without NaCl. This concentration was selected based on preliminary experiments demonstrating substantial root growth inhibition with sufficient genotypic discrimination, consistent with established protocols for wheat salt stress studies [35,36]. The solution was renewed every two days to maintain stable salinity and dissolved oxygen levels. Salt treatment was applied for 5 days prior to tissue sampling. All experiments were conducted with three independent biological replicates of four plants each, yielding 12 plants per genotype per treatment (*n* = 12).

### 2.3. Phenotyping of Root Morphological Traits

Root morphological traits were assessed 5 days after the onset of salt treatment, a time point at which salinity induced clear growth inhibition without causing severe tissue necrosis. Seedlings were carefully removed from the sponge supports, and roots were gently washed with tap water to remove residual medium. Root systems from individual plants were spread in a transparent tray filled with tap water to minimize overlap and scanned using an Epson Expression 12000XL flatbed scanner (Epson, Nagano, Japan) at a resolution of 400 dpi. Scanned images were analyzed using WinRHIZO Pro software (version 2017a; Regent Instruments Inc., Quebec, Canada) with default threshold and root tracking parameters. The following quantitative traits were extracted for each plant: total root length (TRL; cm), surface area (SA; cm^2^), root volume (RV; mm^3^), average root diameter (ARD; mm), and number of root tips (RT; count).

### 2.4. Statistical Analysis of Phenotypic Data

For each accession, three biological replicates were measured, and the mean values of all five root traits (TRL, SA, RV, ARD, and RT) were calculated and used as phenotypic input for GWAS. For the three representative genotypes subjected to detailed physiological comparison, four individual plants were included per replicate (*n* = 12 per genotype per treatment). Descriptive statistics were obtained using Microsoft Excel (Microsoft Corp., Redmond, WA, USA). The normality of trait distributions was assessed using the Ryan–Joiner test in Minitab 14 (Minitab Inc., State College, PA, USA); traits with a statistic close to 1.000 and *p* ≥ 0.05 were considered normally distributed, and those deviating from normality were log- or Box–Cox-transformed as appropriate. Analysis of variance (ANOVA) was conducted in R (version 4.6.0; R Foundation for Statistical Computing, Vienna, Austria) to evaluate the effects of genotype, treatment (control vs. 200 mM NaCl), and their interaction. Broad-sense heritability (*H*^2^) was estimated on an entry-mean basis. To quantify salt tolerance, relative root trait (RRT) values were calculated as the ratio of the trait value under salt stress to that under control conditions (RRT = Value_Salt/Value_Control); these RRT values and genotype means served as phenotypic inputs for GWAS. Post hoc comparisons among genotypes were performed using Tukey’s Honest Significant Difference (HSD) test at *p* < 0.05.

### 2.5. Genotyping and Genome-Wide Association Study (GWAS)

All accessions were genotyped using the Axiom^®^ Wheat Breeder’s Genotyping Array 35K (Thermo Fisher Scientific, Santa Clara, CA, USA). Quality control procedures followed those described for this collection [33]; single-nucleotide polymorphisms (SNPs) with a minor allele frequency (MAF) < 0.05 or a missing rate > 10% were removed, yielding 22,775 high-quality SNPs for downstream analysis. GWAS was performed using the GAPIT3 package (Genomic Association and Prediction Integrated Tool, version 3) in R [37]. Population structure was assessed by principal component analysis (PCA), and the first three principal components (PC1–PC3) were included as covariates to correct for population stratification. Genetic relatedness among accessions was accounted for using a kinship matrix estimated by the VanRaden method [38], as implemented in GAPIT3. Two complementary statistical models were employed: a Mixed Linear Model (MLM) and the Fixed and Random Model Circulating Probability Unification (FarmCPU) model, which iteratively optimizes multiple-loci detection while controlling for population structure [39].

A primary significance threshold of −log_10_*p* ≥ 5.65 was set based on the Bonferroni correction (α = 0.05/22,775 ≈ 2.20 × 10^−6^) [40,41]. A suggestive threshold of −log_10_*p* ≥ 4.36 (*p* ≈ 4.39 × 10^−5^) was additionally applied to retain candidate loci independently supported by RNA-seq transcriptomic evidence, consistent with thresholds widely adopted in wheat GWAS studies [42,43]. SNPs exceeding the suggestive threshold in both MLM and FarmCPU models were considered significant marker-trait associations (MTAs), with priority given to FarmCPU results owing to its higher power for detecting multiple loci simultaneously.

Functional annotations of significant SNPs and candidate genes were retrieved from the Ensembl Plants database (http://plants.ensembl.org) based on the *Triticum aestivum* reference genome (IWGSC RefSeq v1.0). Candidate genes were defined as those located within a ±250 kb window flanking each significant SNP, consistent with reported linkage disequilibrium (LD) decay estimates in Korean wheat germplasm (74.7–393 kb) [34].

### 2.6. Candidate Gene Identification and Haplotype Analysis

Putative candidate genes were identified within a ±250 kb physical window flanking each significant lead SNP, based on the IWGSC RefSeq v1.0 genome annotation. The predicted biological functions and domain architectures of these candidate genes were assessed using the Ensembl Plants (http://plants.ensembl.org), NCBI (https://www.ncbi.nlm.nih.gov), and UniProt (https://www.uniprot.org) databases. Phenotypic differences in root traits and salt-tolerance indices between haplotype groups were statistically evaluated using *t*-tests or ANOVA in R, depending on the number of groups being compared. These haplotype-based categorizations served as the basis for selecting contrasting genotypes (salt-tolerant vs. salt-sensitive) for subsequent RNA-seq transcriptome profiling.

To characterize the structural and functional properties of the candidate gene *TraesCS7B02G418900*, the predicted protein sequence was submitted to InterProScan (v5.77-108.0; InterPro v108.0) [44] for domain annotation. Protein family membership, conserved domain architecture, and associated Gene Ontology (GO) molecular function terms were retrieved from the InterPro database. To evaluate the structural independence of *TaIAO* from previously characterized wheat aldehyde oxidase isoforms, its exon–intron structure and encoded protein length were compared with published wheat *AO* gene models [25]. Gene nomenclature for *TaIAO* followed the guidelines for wheat gene designation established by the international wheat research community [45].

### 2.7. RNA Extraction and Transcriptome Analysis (RNA-Seq)

The three genotypes selected for in-depth analysis (cv. Sarajevo 1, cv. CI 17260, and cv. Keumkang; Section 2.1) were grown in hydroponic culture as described in Section 2.2. After 10 days of growth, plants were subjected to 200 mM NaCl (salt treatment) or tap water (control) for 5 days, and root tissues were collected from three independent biological replicates per genotype per treatment.

Total RNA was extracted from 100 mg of ground root tissue using the Ribospin™ Seed/Fruit Kit (GeneAll Biotechnology, Seoul, Republic of Korea) according to the manufacturer’s instructions, including on-column DNase I treatment to remove genomic DNA. RNA concentration and purity were assessed using a BioDrop µLite+ spectrophotometer (Biochrom, Cambridge, UK), and RNA integrity was verified using an Agilent 2100 Bioanalyzer (Agilent Technologies, Santa Clara, CA, USA).

Sequencing libraries were constructed using the TruSeq Stranded mRNA Library Prep Kit (Illumina, San Diego, CA, USA), according to the manufacturer’s protocol. Libraries were sequenced on an Illumina NovaSeq 6000 platform (Illumina, San Diego, CA, USA) to generate 100-bp paired-end reads.

### 2.8. RNA-Seq Data Processing and Differential Expression Analysis

Raw sequencing reads were processed using Trimmomatic [46] to remove adapter sequences and low-quality bases. High-quality clean reads were aligned to the *Triticum aestivum* reference genome (IWGSC RefSeq v1.0) using HISAT2 [47], and gene expression levels were quantified by counting reads mapped to each gene using feature counts [48]. Differential expression analysis was performed using the edgeR package [49] in R. Prior to statistical modeling, genes with extremely low counts were first filtered out to improve detection power, and read counts were then normalized using the trimmed mean of M-values (TMM) method. Differentially expressed genes (DEGs) were identified based on a significance threshold of false discovery rate (FDR) < 0.05 and an absolute log_2_ fold change (|log_2_FC| > 1). To interpret the biological functions of DEGs, Gene Ontology (GO) enrichment and Kyoto Encyclopedia of Genes and Genomes (KEGG) pathway analyses were performed, and expression patterns were visualized using volcano plots and heatmaps. To prioritize high-confidence candidate genes, genes located within ±250 kb of significant GWAS SNPs were cross-referenced with the DEG list to identify loci supported by both genomic association and differential transcriptional responses to salinity.

### 2.9. Detection of Reactive Oxygen Species (ROS)

For each biological replicate, root tip segments were collected from 20 individual plants per genotype per treatment grown under the conditions described in Section 2.2; three independent biological replicates were prepared per genotype per treatment, yielding a total of 60 plants per genotype per treatment for this assay. To detect reactive oxygen species (ROS) in root cells, root tips were excised from seedlings and transferred to a 10 mM MES-KCl buffer (pH 6.1) for stabilization at room temperature in the dark for 5 min. The root tips were then immersed in 10 mM MES-KCl buffer (pH 6.1) containing 20 μM 2′,7′-dichlorodihydrofluorescein diacetate (H_2_DCFDA) and incubated in the dark for 20 min. After incubation, the roots were washed three times for 5 min each in 10 mM MES-KCl buffer (pH 6.1). ROS accumulation was visualized using a confocal laser scanning microscope at excitation and emission wavelengths of 488 nm and 520 nm, respectively.

### 2.10. Endogenous IAA Measurements

The indole-3-acetic acid (IAA) content in root tissues was quantified using an enzyme-linked immunosorbent assay (ELISA). Root tissues from 20 individual plants per genotype per treatment were pooled and flash-frozen in liquid nitrogen. An aliquot of approximately 100 mg of pooled frozen tissue was weighed and homogenized in phosphate-buffered saline (PBS, pH 7.4) at a sample-to-buffer ratio of 1:9 (*w*/*v*, g/mL). The homogenates were centrifuged at 3000× *g* for 20 min at 4 °C, and the resulting supernatants were collected for analysis. IAA quantification was performed using a Plant Indole-3-Acetic Acid ELISA Kit (SUNLONG, Cat. No. SL0011PI, Hangzhou, China), according to the manufacturer’s protocol, and absorbance was measured at 450 nm. The final IAA content was expressed relative to tissue fresh weight (ng/g FW). All measurements were conducted with three independent biological replicates (*n* = 3 pooled replicates, each derived from 20 individual plants), yielding a total of 60 plants per genotype per treatment.

### 2.11. Quantitative Real-Time PCR (qRT-PCR)

To evaluate the transcriptional responses of candidate genes under salt stress, quantitative real-time PCR (qRT-PCR) was performed for nine genes spanning four functional categories: expansins (*TaExpansin-4A*, *-4B*, *-4D*), PIN-like auxin transporters (*TaPIN-6A*, *-7A*, *-7B*), class III peroxidases (*TaPOD1*, *TaPOD-2D*), and the GWAS-identified locus *TaIAO*. The same three genotypes used for RNA-seq analysis (cv. Keumkang, cv. Sarajevo 1, and cv. CI 17260) were examined under identical treatment conditions (control and 200 mM NaCl for 5 days). For each biological replicate, root tissues were collected from 20 individual plants per genotype per treatment and pooled prior to RNA extraction; three independent biological replicates were prepared per genotype per treatment, yielding a total of 60 plants per genotype per treatment for this assay. Total RNA was extracted from root tissues, as described in Section 2.7, and first-strand cDNA was synthesized using ReverTra Ace™ qPCR RT Master Mix (TOYOBO, Osaka, Japan), according to the manufacturer’s instructions. PCR amplification was carried out on a Rotor-Gene Q instrument (QIAGEN, Hilden, Germany) using a QuantiNova SYBR Green PCR Kit (QIAGEN, Hilden, Germany) according to the manufacturer’s instructions. Each sample was analyzed with three biological and three technical replicates. The TaActin gene (accession AF326781) was used as the internal reference gene for normalization. Relative gene expression levels were calculated using the 2^−ΔΔCT^ method [50]. Primer sequences for all nine target genes, along with their corresponding IWGSC RefSeq v1.0 gene model identifiers and chromosomal assignments, are provided in Supplementary Table S2. The qRT-PCR primer sets for the expansin and PIN-like gene families were designed to target specific homeologous loci on chromosomes 4A, 4B, and 4D, and chromosomes 6A, 7A, and 7B, respectively, which are distinct from the RNA-seq DEG loci (*TaExpansin*: TraesCS1D02G299700 on chromosome 1D; *TaPIN-like*: TraesCSU02G083100 on an unassigned scaffold). The RNA-seq DEG loci were excluded from qRT-PCR primer design because their expression levels were near-zero or undetectable across genotypes under control conditions, precluding reliable genotype-comparative amplification. Instead, homeologous loci with detectable basal expression were selected to enable gene family-level expression analysis across genotypes. This primer design strategy and its implications for data interpretation are addressed in Section 3.7.

### 2.12. Statistical Analysis

Data analysis was performed using IBM SPSS Statistics 27 (IBM Corp., Armonk, NY, USA). Results are presented as mean ± standard error (SE). Differences among group means were evaluated by one-way analysis of variance (ANOVA) or Welch’s *t*-test, where appropriate, and *p*-values were calculated accordingly. Post hoc comparisons were performed using Duncan’s Multiple Range Test. Statistical significance was set at *p* < 0.05.

## 3. Results

### 3.1. Phenotypic Variation and Plasticity in Root Architecture Reveal Distinct Salt-Tolerance Strategies

**Figure 2:**
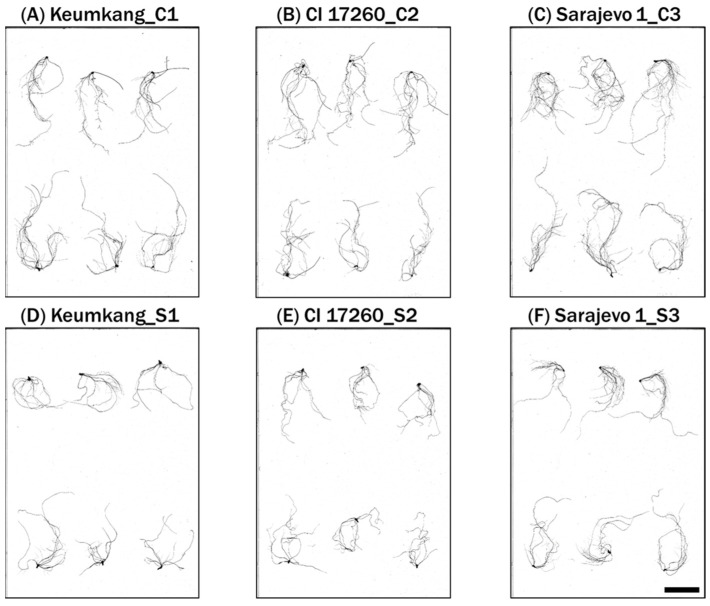
Comparison of root system architecture (RSA) among wheat genotypes under control and salt stress conditions. Representative WinRHIZO scans showing root morphology under control and salt-treated (200 mM NaCl) conditions. (**A**–**C**) Roots grown under control conditions. (**D**–**F**) Roots grown under salt stress. Note the preservation of lateral root density and branching complexity in Sarajevo 1 (**F**) compared with the pronounced architectural reduction in CI 17260 (**E**) under salt stress. Scale bar = 5 cm.

To evaluate genotypic responses to salt stress (200 mM NaCl), root system architecture (RSA) in wheat seedlings was quantitatively assessed using the WinRHIZO scanning system. Morphological comparison between control and salt-treated conditions revealed distinct root phenotypes among the three genotypes (Table 1, Figure 2).

Under control conditions, Sarajevo 1 exhibited the most well-developed root system, with significantly higher total root length (158.7 ± 8.8 cm), surface area (15.5 ± 0.8 cm^2^), and root tip number (277.3 ± 27.6) compared with the other genotypes. The salt-sensitive genotype CI 17260 showed moderate baseline root growth, while the standard cultivar Keumkang displayed intermediate values (Table 1).

Salt stress induced differential reductions in root traits across genotypes, with significant genotypic variation in tolerance (ANOVA, *p* < 0.05). Sarajevo 1 maintained 95.2% of its total root length (151.0 ± 7.0 cm) and exhibited a 13.3% increase in root volume relative to the control (113.3% in control). The relative root volume retention of Sarajevo 1 was significantly higher than that of other genotypes (Table 1, Tukey’s HSD, *p* < 0.05). Representative WinRHIZO images (Figure 2C,F) show that Sarajevo 1 preserved a dense, highly branched root system with extensive lateral roots under saline conditions, compared with the sparse architecture observed in CI 17260.

Conversely, CI 17260 exhibited the greatest reductions in root traits under salt stress. Root length and root tip number declined markedly, and salt-treated seedlings (Figure 2B,E) displayed a thin and sparse root system with substantially reduced branching density relative to the controls. The standard cultivar Keumkang demonstrated intermediate salt tolerance, retaining a lesser proportion of root length and volume than Sarajevo 1 (Table 1). Visual comparison (Figure 2A,D) indicated that Keumkang preserved core lateral branching structures to a greater extent than CI 17260, although the quantitative reduction in root traits was more pronounced than in Sarajevo 1. Shoot growth across all three genotypes was recorded at the time of root tissue sampling (Supplementary Figure S2). Under control conditions, all three genotypes produced well-developed seedlings with fully expanded leaves. Seedlings subjected to 200 mM NaCl for five days showed apparent reductions in shoot growth relative to control conditions, with the degree of suppression corresponding to the tolerance ranking established by root trait analysis (CI 17260 < Keumkang < Sarajevo 1).

### 3.2. Genome-Wide Association Mapping Identifies Genomic Loci and Candidate Genes Linking Auxin Biosynthesis to Root Adaptation

**Figure 3:**
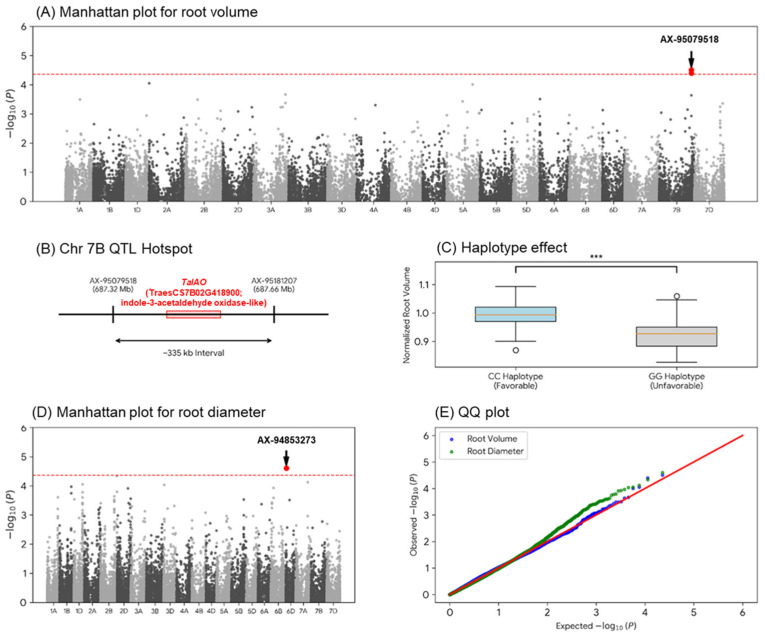
Genome-wide association study (GWAS) results identifying genomic loci and candidate genes associated with root adaptation under salt stress. (**A**) Manhattan plot for root volume. The arrow indicates the association peak on chromosome 7B identified by the FarmCPU model. The red horizontal dashed line represents the suggestive threshold (−log_10_*p* ≥ 4.36), applied to identify candidate genomic regions associated with root adaptation under salt stress while controlling for false positives within the FarmCPU framework. (**B**) Schematic representation of the chromosome 7B QTL interval. The candidate gene *TaIAO* (*TraesCS7B02G418900*; indole-3-acetaldehyde oxidase-like) is physically located between the two significant SNPs, AX-95079518 and AX-95181207, defining a ~335 kb physical interval, consistent with the ± 250 kb LD decay window applied for candidate gene identification in this population. (**C**) Haplotype effect analysis of the peak SNP AX-95079518. Boxplots show phenotypic differences in normalized root volume between the favorable CC haplotype (associated with the tolerant genotype Sarajevo 1) and the unfavorable GG haplotype (associated with the sensitive genotype CI 17260). Asterisks (***) indicate a statistically significant difference (*p* < 0.001). (**D**) Manhattan plot for average root diameter, showing an association signal on chromosome 6D (SNP AX-94853273). The red horizontal dashed line represents the suggestive threshold (−log_10_*p* ≥ 4.36). (**E**) Quantile–Quantile (QQ) plots for root volume (blue) and root diameter (green). Deviation of observed *p*-values from the expected distribution (red line) at the upper tail indicates putative associations rather than population structure artifacts.

To identify genomic loci underlying variation in root plasticity under salt stress, GWAS was performed on 566 wheat accessions. Normalized root volume data (NaCl/Control ratio) were used as the primary phenotype, and the FarmCPU model was employed to control for population structure and kinship. The statistical reliability of the analysis was confirmed by quantile–quantile (QQ) plots, demonstrating adequate control of type I errors and genomic inflation (Figure 3E).

The analysis identified a candidate quantitative trait locus (QTL) for root volume maintenance under salt stress on chromosome 7B (Figure 3A, Table 2). Two SNPs consistently exceeded the suggestive threshold (−log_10_*p* ≥ 4.36); the peak SNP, AX-95079518 (687,324,247 bp; FarmCPU −log_10_*p* = 4.50, *p* = 3.14 × 10^−5^) and AX-95181207 (687,659,784 bp; −log_10_*p* = 4.40). The physical distance between these SNPs spans ~335 kb, consistent with the ±250 kb LD decay window applied for candidate gene identification. Within this interval, *TaIAO* (*Triticum aestivum* Indole-3-Acetaldehyde Oxidase-like; *TraesCS7B02G418900*) was identified as the primary candidate gene, based on its annotation as a predicted participant in tryptophan-dependent auxin biosynthesis.

TaIAO (*TraesCS7B02G418900*; Chr 7B: 687,419,612–687,433,035 bp) is situated approximately 95 kb downstream of AX-95079518 and 227 kb upstream of AX-95181207 (Figure 3B), supporting its positional candidacy. To characterize the structural properties of the encoded protein, the predicted 867-aa sequence was subjected to domain annotation using InterProScan (v5.77-108.0; InterPro v108.0) [44]. The analysis identified membership in the Aldehyde oxidase/xanthine dehydrogenase-like superfamily (IPR016208), with three canonical catalytic domains identified: an N-terminal 2Fe-2S ferredoxin domain, a central FAD-binding domain, and a C-terminal Moco-binding domain. Associated GO Molecular Function terms included iron ion binding (GO:0005506), oxidoreductase activity (GO:0016491), iron-sulfur cluster binding (GO:0051536), 2 iron, 2 sulfur cluster binding (GO:0051537), and flavin adenine dinucleotide (FAD) binding (GO:0050660). Structural comparison further revealed that *TaIAO* (867 aa; 6-exon structure) is architecturally distinct from the wheat AO-B3 isoform (~1262 aa; 10-exon structure) characterized by Colasuonno et al. [25], indicating that *TraesCS7B02G418900* represents an independently annotated locus within the wheat AO gene family. The designation *TaIAO* reflects its substrate specificity for indole-3-acetaldehyde oxidation (EC 1.2.3.7) and follows the IWGSC wheat gene nomenclature guidelines [45].

Haplotype analysis of the peak SNP AX-95079518 revealed that accessions carrying the favorable CC haplotype (major allele frequency = 67.7%, including Sarajevo 1) exhibited significantly higher root volume retention than those carrying the GG haplotype (minor allele frequency = 32.3%, including CI 17260) (*p* < 0.001; Figure 3C). The negative effect size (β = −0.077) indicates that the minor G allele is associated with reduced root volume retention. In addition, a trait-specific association for average root diameter was identified on chromosome 6D (SNP AX-94853273; FarmCPU −log_10_*p* = 4.60, *p* = 2.51 × 10^−5^; Figure 3D, Table 2), with no significant association with root volume, confirming the trait specificity of this locus. Gene annotation linked this SNP to *TraesCS6D02G047000*, encoding an F-box domain-containing protein.

These associations were cross-validated were cross-validated using an MLM. The lead SNP AX-95079518 on chromosome 7B remained the most significant marker for root volume (*p* = 4.49 × 10^−5^; Supplementary Table S1), and AX-94853273 on chromosome 6D was confirmed as a top-ranking marker for root diameter (*p* = 5.36 × 10^−5^; Table 2, Supplementary Table S1). QQ and Manhattan plots (Supplementary Figure S3) support that the detected loci represent association signals rather than population structure artifacts.

### 3.3. Genotype-Specific Transcriptomic Profiles Reveal Divergent Gene Regulation Patterns Under Salt Stress

To characterize the global transcriptomic response to salt stress, the number and distribution of differentially expressed genes (DEGs) were analyzed across the three genotypes (Table 3, Supplementary Figure S4). Distinct transcriptomic patterns were observed among genotypes. Keumkang (Supplementary Figure S4C) exhibited a predominant upregulation of genes (9069 upregulated vs. 8280 downregulated genes). Sarajevo 1 (Supplementary Figure S4A) showed a pronounced shift toward downregulation (10,533 downregulated vs. 7883 upregulated genes). CI 17260 (Supplementary Figure S4B) displayed a relatively balanced response (8246 upregulated vs. 7909 downregulated genes).

**Figure 4:**
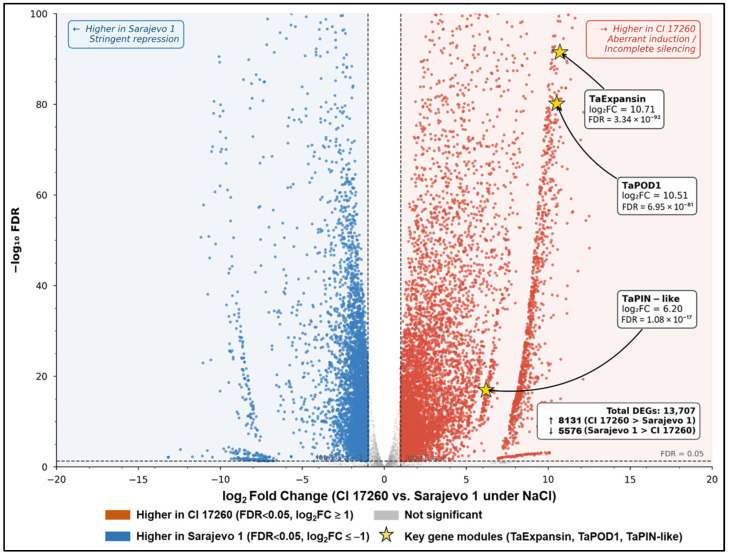
Divergent regulation of key gene modules under salt stress. Volcano plot displaying differential gene expression between the salt-sensitive genotype (CI 17260) and the salt-tolerant genotype (Sarajevo 1) under salt stress (Comparison 4). Gold star symbols (★) in the upper-right quadrant indicate genes expressed at substantially higher levels in CI 17260 relative to Sarajevo 1: (1) *TaExpansin* (log_2_FC = 10.71 in Comparison 4; aberrantly induced under stress from basal levels) and (2) *TaPIN-like* (log_2_FC = 6.20 in Comparison 4; aberrantly induced under stress from basal levels). *TaPOD1* (log_2_FC = 10.51 in Comparison 4; incompletely repressed, residual ~199 reads in CI 17260) indicates incomplete transcriptional repression in CI 17260 under stress, in contrast to the complete transcriptional shutdown observed in Sarajevo 1 (read counts = 0 across all biological replicates).

Direct genotypic comparison under salt stress (Comparison 4; 13,707 total DEGs) revealed marked divergence in the regulation of genes associated with root growth and defense mechanisms (Figure 4; Table 4). Three functionally distinct gene modules showed substantially divergent expression between the two genotypes. A cell wall-loosening factor (*TaExpansin*, *TraesCS1D02G299700*; Comp 4 log_2_FC = 10.71, FDR = 3.34 × 10^−92^) and an auxin transporter (*TaPIN-like*, *TraesCSU02G083100*; Comp 4 log_2_FC = 6.20, FDR = 1.08 × 10^−17^) were expressed at substantially higher levels in CI 17260 relative to Sarajevo 1 under salt stress; transcript levels of both genes were near-undetectable in Sarajevo 1 under the same conditions. Regarding ROS scavenging-related transcripts, both genotypes exhibited comparable *TaPOD1* (*TraesCS7D02G417400*) expression under control conditions (~2000 reads). Under stress conditions, however, Sarajevo 1 showed a complete transcriptional shutdown (read counts = 0 across all biological replicates; Comp 1 log_2_FC = −13.60, FDR = 1.55 × 10^−287^), while CI 17260 maintained partial residual expression (~199 reads; Comp 2 log_2_FC = −2.89, FDR = 5.86 × 10^−104^). This divergent regulation resulted in a substantial relative difference between the two genotypes under stress (*TaPOD1*, Comp 4 log_2_FC = 10.51; Figure 4; Table 4).

Collectively, these transcriptomic profiles indicate that CI 17260 maintained or induced expression of specific growth- and defense-related genes under salt stress, whereas Sarajevo 1 exhibited a pronounced global downregulation of the same modules (Supplementary Figure S4A; Figure 4).

### 3.4. Integration of GWAS and RNA-Seq Identifies Divergent Candidate Gene Modules Under Salt Stress

**Figure 5:**
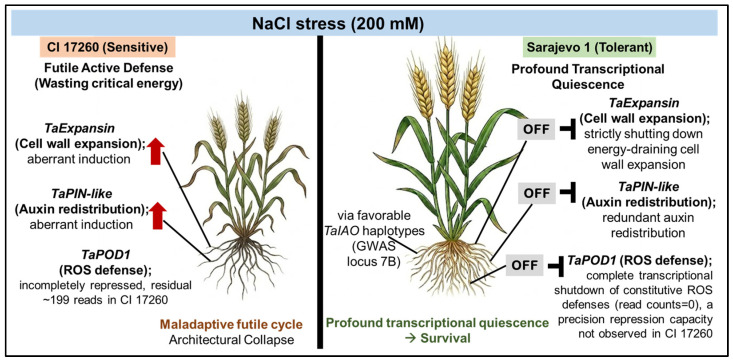
Conceptual model of divergent root adaptive strategies under salt stress: transcriptional quiescence in the tolerant genotype vs. transcriptional dysregulation in the sensitive genotype. The model depicts divergent patterns of gene network regulation in the root system under 200 mM NaCl. The tolerant genotype Sarajevo 1 (**right**) exhibits a pre-established root system associated with favorable *TaIAO* haplotypes at the chromosome 7B GWAS locus and under osmotic stress shows complete transcriptional repression of cell wall expansion (*TaExpansin*), auxin redistribution (*TaPIN-like*), and constitutive ROS defenses (*TaPOD1*; read counts = 0), a pattern of coordinated repression absent in CI 17260. In contrast, the sensitive genotype CI 17260 (**left**) aberrantly induces or fails to completely repress these modules under stress, a pattern associated with energetically costly transcriptional dysregulation and severe root architectural deterioration.

Integration of GWAS and RNA-Seq results identified a set of functionally distinct candidate genes exhibiting markedly divergent expression patterns between Sarajevo 1 and the sensitive genotype CI 17260 under salt stress (Figure 5; Table 5). At the genetic level, the most significantly associated GWAS locus on chromosome 7B harbored *TaIAO* (*TraesCS7B02G418900*), encoding an indole-3-acetaldehyde oxidase-like protein. Transcript levels of this gene in both genotypes under salt stress fell below the statistical detection threshold for DEGs (Table 5). Under non-stress conditions, Sarajevo 1 exhibited substantially greater total root length (158.7 ± 8.8 cm) and root tip number (277.3 ± 27.6) than CI 17260 (90.2 ± 4.5 cm and 93.3 ± 7.9, respectively; both *p* < 0.001, Tukey’s HSD, Table 1).

At the transcriptional level, three gene modules showed pronounced differential expression in Comparison 4 (Table 5). A cell wall-loosening factor (*TaExpansin*, TraesCS1D02G299700; log_2_FC = 10.71, FDR = 3.34 × 10^−92^), an auxin efflux carrier (*TaPIN-like*, TraesCSU02G083100; log_2_FC = 6.20, FDR = 1.08 × 10^−17^), and a Class III peroxidase (*TaPOD1*, TraesCS7D02G417400; log_2_FC = 10.51, FDR = 6.95 × 10^−81^) were all expressed at significantly higher levels in CI 17260 than in Sarajevo 1 under salt stress (Table 5; Figure 5). In Sarajevo 1, transcript levels of *TaExpansin* and *TaPIN-like* were near-zero under stress, and *TaPOD1* underwent a complete transcriptional shutdown (read counts = 0 across all biological replicates). The divergent regulation of these modules between the two genotypes is conceptually illustrated in Figure 5.

### 3.5. Endogenous IAA Quantification Reveals Genotype-Specific Dynamics in Auxin Homeostasis Under Salt Stress

To directly assess endogenous auxin levels across genotypes, the indole-3-acetic acid (IAA) content was quantified in root tissues of all three genotypes under control and salt stress conditions using ELISA (Table 6). Under control conditions, a statistically significant difference in root IAA content was detected among the three genotypes (one-way ANOVA: F(2,6) = 5.52, *p* = 0.044). Specifically, Sarajevo 1 (112.8 ± 5.0 ng/g FW, Duncan group a) and Keumkang (109.2 ± 5.0 ng/g FW, Duncan group a) exhibited significantly higher constitutive IAA levels than CI 17260 (93.7 ± 2.4 ng/g FW, Duncan group b). Under 200 mM NaCl treatment, contrasting IAA dynamics were observed between the salt-tolerant and salt-sensitive genotypes. In Sarajevo 1, root IAA content decreased significantly from 112.8 ± 5.0 to 91.1 ± 3.9 ng/g FW (Welch’s *t*-test: t = 3.40, *p* = 0.030), representing a reduction of approximately 19.2% relative to its own control value. In contrast, CI 17260 exhibited a significant increase in root IAA content under salt stress, rising from 93.7 ± 2.4 to 104.5 ± 2.6 ng/g FW (t = −3.08, *p* = 0.037), an increase of approximately 11.6% relative to its control. Keumkang showed no significant change under salt stress (109.2 ± 5.0 vs. 103.8 ± 0.9 ng/g FW; t = 1.07, *p* = 0.392).

Consequently, the genotypic ranking in root IAA content was inverted under salt stress relative to control conditions. Under saline conditions, CI 17260 (104.5 ± 2.6 ng/g FW, Duncan group a) and Keumkang (103.8 ± 0.9 ng/g FW, Duncan group a) no longer differed significantly from each other but were both significantly higher than Sarajevo 1 (91.1 ± 3.9 ng/g FW, Duncan group b; one-way ANOVA: *F*(2,6) = 7.45, *p* = 0.024). These results indicate that Sarajevo 1 underwent a stress-associated reduction in root IAA content, whereas CI 17260 showed a significant increase under equivalent salt stress conditions. The biological implications of these contrasting IAA dynamics are discussed in Section 4.2.

### 3.6. Salt Stress Attenuates H_2_DCFDA Fluorescence in Root Tips of All Three Wheat Genotypes

To assess reactive oxygen species (ROS) accumulation in root tips, H_2_DCFDA fluorescence imaging was performed on intact root tip segments excised from seedlings of all three genotypes under control and salt stress conditions (Supplementary Figure S5). Under control conditions, green fluorescence indicative of intracellular ROS was detected in the root tips of all three genotypes, consistent with basal ROS levels in root tip cells. Following a five-day exposure to 200 mM NaCl, the fluorescence intensity was substantially reduced across all three genotypes relative to control conditions, with low or undetectable signals in the salt-stressed samples.

At the qualitative level of this assay, no pronounced genotype-specific difference in the direction or magnitude of fluorescence attenuation was apparent under salt stress, although baseline fluorescence intensities under control conditions appeared to vary modestly among genotypes. These results therefore provide qualitative evidence for reduced ROS signals in root tips under severe salt stress, but do not permit a direct quantitative comparison of ROS levels between contrasting genotypes, as quantitative fluorometric analysis was not performed. The mechanistic basis for this fluorescence attenuation cannot be conclusively established from the present qualitative imaging data alone; possible explanations include reduced ROS-generating metabolic activity associated with meristematic quiescence under severe osmotic stress, enhanced antioxidant scavenging capacity, or altered dye uptake resulting from changes in membrane permeability. The relationship between the transcriptional repression of *TaPOD1* and *TaPOD-2D* observed in Sarajevo 1, and the broader pattern of reduced fluorescence common to all genotypes is discussed in Section 4.3.

### 3.7. Quantitative RT-PCR Analysis of Candidate Gene Expression Under Salt Stress

To examine the expression of candidate genes identified by RNA-seq analysis and the GWAS-identified candidate locus *TaIAO* using an independent quantification approach, quantitative real-time PCR (qRT-PCR) was performed for nine genes across four functional categories: expansins (*TaExpansin-4A*, *-4B*, *-4D*), PIN-like auxin transporters (*TaPIN-6A*, *-7A*, *-7B*), class III peroxidases (*TaPOD1*, *TaPOD-2D*), and *TaIAO*. Primer sequences and corresponding gene identifiers are listed in Supplementary Table S2. Statistical significance of within-genotype CK-to-salt comparisons was evaluated by one-way ANOVA with post hoc comparison or Welch’s *t*-test, as appropriate (Section 2.12). Expression ratios normalized to TaActin are shown in Supplementary Figure S6. The chromosomal correspondence between qRT-PCR target loci and RNA-seq DEGs, together with the genotype-specific expression results, is summarized in Supplementary Table S3.

Expansin family members. *TaExpansin-4A* (TraesCS4A02G339200) showed no statistically significant change under salt treatment in any genotype (all comparisons: *p* > 0.05). *TaExpansin-4B* (TraesCS4B02G271300) showed a significant decrease in expression in Sarajevo 1 under salt stress (*p* < 0.05), with no significant change in Keumkang or CI 17260 (*p* > 0.05 for both). *TaExpansin-4D* (TraesCS4D02G270500) likewise showed a significant decrease in Sarajevo 1 (*p* < 0.01), with no significant change in Keumkang or CI 17260 (*p* > 0.05 for both). The significant downregulation of *TaExpansin-4B* and *TaExpansin-4D* in Sarajevo 1 under salt stress is notable in the context of the RNA-seq finding of expansin suppression in the tolerant genotype (TraesCS1D02G299700, chromosome 1D; Section 3.3), although the qRT-PCR primers used here target genes on chromosomes 4A, 4B, and 4D at chromosomal loci distinct from the RNA-seq-identified expansin DEG.

PIN-like auxin transporter family members. None of the three PIN-like family members examined—*TaPIN-6A* (TraesCS6A02G308600), *TaPIN-7A* (TraesCS7A02G492400), and *TaPIN-7B* (TraesCS7B02G398100)—showed statistically significant changes in expression under salt treatment in any genotype (all comparisons: *p* > 0.05).

Class III peroxidase family members. *TaPOD1* (TraesCS7D02G417400) showed a significant decrease in expression under salt treatment in Keumkang (*p* < 0.05), whereas no significant change was observed in Sarajevo 1 or CI 17260 (*p*> 0.05 for both). It is noted that the qRT-PCR result for *TaPOD1* is not fully concordant with the RNA-seq finding, in which near-complete transcriptional repression of this gene was identified in Sarajevo 1 under salt stress (Comparison 1: log_2_FC = −13.60, FDR = 1.55 × 10^−287^; Section 3.3); this discordance is further addressed in Section 4.3. *TaPOD-2D* (TraesCS2D02G511200) also showed a significant decrease in expression under salt stress in Keumkang (*p* < 0.01), with no significant change in Sarajevo 1 or CI 17260 (*p* > 0.05 for both). Basal *TaPOD-2D* expression levels under control conditions were approximately 17–18-fold higher in Sarajevo 1 and CI 17260 than in Keumkang (mean expression ratio ≈ 18, ≈17, and ≈1, respectively).

TaIAO expression. *TaIAO* (*TraesCS7B02G418900*) showed a significant decrease in expression under salt treatment in Keumkang (*p* < 0.01), whereas no significant change was observed in Sarajevo 1 or CI 17260 (*p* > 0.05 for both). In the RNA-seq analysis, TaIAO transcript levels did not meet the threshold criteria for DEG classification (FDR < 0.05 and |log_2_FC| ≥ 1) in any genotype. This genotype-specific pattern of *TaIAO* expression under salt stress, in which a significant decrease was detected only in Keumkang and not in Sarajevo 1 or CI 17260, is further discussed in the context of the proposed role of this locus in Section 4.2.

Taken together, the qRT-PCR analysis of nine candidate genes revealed that statistically significant responses under salt stress were detected predominantly in Keumkang (*TaPOD1*, *p* < 0.05; *TaPOD-2D*, *p* < 0.01; *TaIAO*, *P* < 0.01) and in Sarajevo 1 for the expansin family members on chromosomes 4B and 4D (*TaExpansin-4B*, *p* < 0.05; *TaExpansin-4D*, *p* < 0.01), whereas no significant changes were detected in CI 17260 for any of the nine genes examined. The qRT-PCR result for TaPOD1 is not fully concordant with the RNA-seq finding, in which near-complete transcriptional repression was identified in Sarajevo 1 (Comparison 1: log_2_FC = −13.60, FDR = 1.55 × 10^−287^; Section 3.3); this discordance is further addressed in Section 4.3.

## 4. Discussion

### 4.1. Integrating Genetic Architecture and Stress-Responsive Networks: A Two-Tier Model of Root Adaptation

Soil salinity represents a major environmental constraint that severely limits global wheat productivity, primarily by disrupting the soil–root–shoot continuum and imposing osmotic stress, ionic toxicity, and oxidative damage [6,9,10]. The phenotypic plasticity of the root system architecture (RSA) has therefore emerged as an important adaptive strategy by which plants optimize resource acquisition under heterogeneous saline environments [16,51]. In this study, we integrated high-resolution root phenotyping across 566 wheat accessions, genome-wide association study (GWAS), and comparative transcriptomic profiling to dissect the genetic and molecular determinants of RSA adaptation under salt stress.

Our complementary experimental design enabled us to distinguish between constitutive genetic factors and stress-responsive transcriptional programs that collectively govern root plasticity. Our data support a two-tier regulatory model [52] in which genetic variation at the *TaIAO* locus (chromosome 7B) establishes differential basal auxin biosynthetic capacity among genotypes, and this pre-existing genetic architecture subsequently influences the efficacy of the stress-induced deployment of downstream auxin–ROS signaling networks. Specifically, these stress-responsive networks encompass three core modules whose precise transcriptional control, rather than stress-induced activation alone, appears to underlie the adaptive capacity of the tolerant genotype: polar auxin redistribution (PIN-like transporters) [18], constitutive ROS defense (Class III peroxidases), and cell wall loosening (expansins). The stress-responsive regulation of each module has been documented in wheat and related cereals: overexpression of *TaPRX-2A* (Class III peroxidase) [28] and *TaEXPA7-B* (*Expansin*) [31] confers improved salinity tolerance, underscoring the importance of precise dosage control of these modules in adaptive responses.

The biological robustness of our transcriptomic findings is supported by highly stringent statistical thresholds (FDR < 0.05) and notable phenotype–transcriptome concordance. Our comparative RNA-Seq analysis showed that *TaExpansin*, *TaPOD1* (Class III peroxidase), and *TaPIN-like* were all expressed at significantly higher levels in CI 17260 than in the tolerant Sarajevo 1 under salt stress (Comp 4; log_2_FC = 10.71, 10.51, and 6.20, respectively), whereas Sarajevo 1 underwent complete transcriptional repression of *TaPOD1* (read counts = 0) and near-complete repression of *TaExpansin* and *TaPIN-like*; the molecular basis of this divergent regulation is discussed in Section 4.3. Notably, Sarajevo 1 harbors *TaIAO* haplotypes (CC at SNP AX-95079518) associated with superior root architectural complexity even under control conditions. This integrated framework is consistent with the pronounced root architectural preservation observed in Sarajevo 1 under 200 mM NaCl (Table 1), in contrast to the severe growth inhibition observed in CI 17260.

### 4.2. The Constitutive Genetic Basis for Salt-Responsive Transcriptional Plasticity

A central and seemingly paradoxical finding of this study is that *TaIAO*, a candidate gene associated with root volume maintenance under salt stress (−log_10_*p* = 4.50), was not identified as a differentially expressed gene in our RNA-Seq analysis; transcript abundance at this locus consistently remained below the statistical threshold for stress inducibility. Rather than undermining the genetic validity of this association, this dissociation between GWAS significance and transcriptional inducibility supports a two-tier regulatory architecture in which constitutive genetic variation establishes the physiological foundation upon which stress-responsive transcriptional programs are subsequently deployed. This framework is increasingly recognized in plant stress biology, in which genetic polymorphisms conferring abiotic stress tolerance often operate through the modulation of baseline physiological capacity rather than through stress-induced transcriptional cascades [53,54]. This interpretation is further corroborated by qRT-PCR analysis (Section 3.7), in which *TaIAO* showed no significant salt-responsive expression change in either Sarajevo 1 or CI 17260 (*p* > 0.05), consistent with the RNA-seq non-DEG classification and supporting a constitutive rather than stress-inducible mode of action.

The biological rationale for maintaining constitutive auxin biosynthetic capacity is grounded in the indispensable role of auxin homeostasis in coordinating both developmental growth and stress adaptation [55]. In this context, the GWAS-identified candidate gene *TraesCS7B02G418900* encodes a *TaIAO* protein predicted to catalyze the final oxidative step in the tryptophan-dependent auxin biosynthesis pathway—the conversion of indole-3-acetaldehyde (IAAld) to bioactive indole-3-acetic acid (IAA)—positioning this locus as a candidate determinant of *de novo* IAA production capacity.

The structural competence and enzymatic integrity of the *TaIAO*-encoded protein are supported by InterProScan domain analysis (v5.77-108.0; InterPro v108.0) [44], which confirmed the tripartite catalytic architecture comprising an N-terminal 2Fe-2S ferredoxin domain, a central FAD-binding domain, and a C-terminal Moco-binding domain (IPR016208) required for substrate oxidation in the 867-aa protein. This highly conserved domain organization is the hallmark of biochemically characterized plant aldehyde oxidases that catalyze the terminal oxidative step of the tryptamine branch of tryptophan-dependent auxin biosynthesis [56]. Notably, *TaIAO* (867 aa; 6-exon architecture) displays a distinct structural profile from the grain-associated wheat AO3 isoforms (~1262 aa; 10-exon structure) previously characterized by Colasuonno et al. [25]. While those isoforms are primarily linked to abscisic aldehyde oxidation (EC 1.2.3.1) and carotenoid catabolism in the grain, our findings suggest that *TraesCS7B02G418900* represents an independently annotated genomic locus potentially specialized for auxin biosynthesis in vegetative root tissues. In *Arabidopsis*, quantitative measurements of IAA biosynthesis rates using deuterium-labeling combined with GC-SRM-MS analysis of spatially resolved root sections have shown that de novo IAA biosynthesis is highest in the most apical meristematic region of the primary root tip and in the tips of emerging lateral roots [57]. Cell-type-specific quantification has further identified a distinct IAA concentration maximum in the quiescent center of the root apex, with all measured cell types displaying substantial local IAA synthesis capacity [58]. The aldehyde oxidase-like domain architecture of *TaIAO* (IPR016208), predicted to catalyze the terminal oxidative step of the tryptamine branch of auxin biosynthesis (IAAld → IAA; EC 1.2.3.7), is consistent with a potential constitutive role in these meristematic root zones. Although direct biochemical validation of *TaIAO* substrate specificity (EC 1.2.3.7) remains to be performed, indirect physiological support for the proposed *TaIAO*–auxin homeostasis relationship was obtained through ELISA-based IAA quantification in root tissues (Section 3.5; Table 6). The nomenclature *TaIAO* reflects its predicted specificity for indole-3-acetaldehyde oxidation (EC 1.2.3.7)—in contrast to the ABA-related substrate specificity (EC 1.2.3.1) attributed to earlier AO3 models—and follows the principles of the updated IWGSC wheat gene nomenclature guidelines [45].

Under salt stress, plants do not primarily rely on *de novo* auxin biosynthesis to elevate auxin levels; rather, they dynamically redistribute endogenous steady-state IAA pools through modulation of transport polarity and downstream signaling sensitivity [55,59]. Furthermore, Cackett et al. [60] demonstrated that elevated auxin levels observed under saline conditions arise predominantly through altered IAA metabolism and transport rather than enhanced biosynthetic gene expression, and that enlarging baseline IAA pools confers marked improvements in salt tolerance. These findings parallel our observation that favorable *TaIAO* haplotypes confer superior root architecture even under control conditions, suggesting that the CC genotype at SNP AX-95079518 may establish a higher constitutive auxin biosynthetic capacity, potentially supporting more effective stress-responsive redistribution.

This interpretation is robustly supported by our phenotypic data. Under non-stress conditions, Sarajevo 1 exhibited a significantly greater total root length (158.7 ± 8.8 cm) and root tip number (277.3 ± 27.6) than CI 17260 (90.2 ± 4.5 cm and 93.3 ± 7.9 tips, respectively; both *p* < 0.001, Tukey’s HSD test). This pre-existing architectural advantage, consistent with the functional consequences of the favorable *TaIAO* haplotype, provides a larger root system with greater exploratory capacity and more numerous meristematic zones, both critical for maintaining root function under combined osmotic and ionic stress. Such pre-stress architectural differences align with the concept of ‘hidden adaptations’ embedded within constitutive genetic diversity, which may afford plants enhanced resilience to abiotic pressures without requiring *de novo* transcriptional activation [53].

The evolutionary logic underlying the constitutive rather than inducible nature of auxin biosynthesis aligns with analogous principles of plant defense economics [54]. Constitutive traits are evolutionarily favored when selective pressure is frequent or when such traits provide continuous fitness benefits independent of environmental challenge. Since auxin is indispensable for basal root development, natural selection is expected to favor genetic variation that modulates baseline biosynthetic capacity. This contrasts with energetically costly stress-response components such as Class III peroxidases (e.g., *TaPOD1*) and cell wall remodeling proteins (e.g., expansins), whose high constitutive expression would impose substantial metabolic costs under non-stress conditions. The integration of constitutive genetic determinants (*TaIAO* haplotypes) with tightly regulated stress-responsive transcriptional repression, thereby constituting the auxin–ROS regulatory module described in this study, may represent an adaptive strategy favoring metabolic economy by balancing the energetic costs of stress defense against the benefits of phenotypic plasticity.

While previous studies have primarily focused on auxin signaling components, most notably ARFs and Aux/IAA transcriptional repressors, as the principal mediators of salt stress-induced root responses [61,62], the identification of an AO-encoding locus (*TaIAO*) at the chromosome 7B QTL hotspot positions auxin biosynthetic capacity as a potentially underappreciated constitutive determinant of root architectural plasticity. Collectively, these findings suggest that genotypic variation in salt-adaptive root plasticity may partly reflect differences in the intrinsic capacity for *de novo* IAA production, rather than solely differences in downstream signaling sensitivity to endogenous auxin pools. To directly evaluate this interpretation, ELISA was performed across all three genotypes under both conditions (Section 3.5; Table 6). Under non-stress conditions, Sarajevo 1 and Keumkang exhibited significantly higher constitutive IAA levels than CI 17260, consistent with the hypothesis that the favorable CC haplotype at SNP AX-95079518 confers constitutively higher auxin biosynthetic output. Under salt stress, Sarajevo 1 underwent a significant reduction in root IAA content (*p* = 0.030), whereas CI 17260 exhibited a significant increase (*p* = 0.037), resulting in an inversion of the genotypic ranking observed under control conditions. This divergent IAA dynamic is consistent with a potentially dysregulated auxin accumulation pattern in CI 17260 and further supports the proposed two-tier regulatory model. These ELISA data are, however, correlative in nature; direct biochemical characterization of *TaIAO* substrate specificity (EC 1.2.3.7) and in planta functional validation will be required to establish a causal mechanistic link between this locus and constitutive auxin biosynthesis. Furthermore, future studies should employ histochemical or immunolocalization approaches, such as DR5-based auxin-responsive reporters or anti-IAA immunostaining of root cross-sections, to directly map auxin distribution across root tissue zones in contrasting wheat genotypes, providing the spatial resolution that bulk-tissue ELISA quantification cannot achieve.

### 4.3. Divergent Transcriptional Regulation of Auxin–ROS Modules: Mechanistic Basis of Contrasting Salt Tolerance

In addition to the constitutive auxin biosynthesis mediated by the chromosome 7B QTL (*TaIAO* locus), our GWAS identified a trait-specific association for root diameter on chromosome 6D (SNP AX-94853273), mapped to the candidate gene *TraesCS6D02G047000* encoding an F-box domain-containing protein (Table 2). F-box proteins function as the substrate-recognition subunits of the SCF ubiquitin–ligase complex, and, in the auxin signaling context, well-characterized members such as the TIR1/AFB subfamily serve as auxin co-receptors that facilitate Aux/IAA repressor degradation in an auxin-dependent manner, thereby releasing ARFs from inhibition and activating downstream auxin-responsive transcription [63]. *TraesCS6D02G047000* harbors an F-box-like domain superfamily (SSF81383; IPR036047) but lacks an identifiable LRR repeat domain, indicating that its precise subfamily assignment awaits comprehensive phylogenetic characterization. The identification of this F-box locus on chromosome 6D suggests a possible role in modulating auxin signaling sensitivity, potentially contributing to the localized and rapid activation required to adjust root diameter dynamics under osmotic stress. Taken together, the co-identification of these two loci suggests a coordinated regulatory architecture in which constitutive auxin biosynthesis (Chr 7B QTL, *TaIAO*) and auxin signaling-related protein turnover (Chr 6D QTL, F-box) may act in concert to fine-tune root architectural responses under salt stress. The stress-responsive transcriptional layer of the two-tier regulatory architecture is characterized by markedly divergent regulation of three functionally interconnected gene modules: *TaPOD1* (ROS scavenging), *TaPIN-like* (polar auxin redistribution), and *TaExpansin* (cell wall remodeling). Crucially, the key distinction between the tolerant Sarajevo 1 and the sensitive CI 17260 lies not simply in stress-induced activation, but rather in the capacity for precise transcriptional repression of these modules under stress.

*TaPOD1* (TraesCS7D02G417400), a class III peroxidase, exhibited comparable constitutive expression in both genotypes under non-stress conditions (~2000 reads). Under salt stress, however, Sarajevo 1 underwent complete transcriptional shutdown (read counts = 0 across all biological replicates; Comp 1 log_2_FC = −13.60, FDR = 1.55 × 10^−287^), while CI 17260 maintained partial residual expression (~199 reads; Comp 2 log_2_FC = −2.89, FDR = 5.86 × 10^−104^). The undetectable expression level of *TaPOD1* in Sarajevo 1 may provide two potential adaptive advantages under severe osmotic stress. First, it may reduce the biosynthetic and maintenance costs associated with constitutive peroxidase synthesis and the associated NADPH-consuming H_2_O_2_ processing cycles, thereby potentially redirecting resources toward osmotic adjustment and ion compartmentalization. Second, although Class III peroxidases function as ROS scavengers, their stress-induced overactivity can paradoxically promote ROS-mediated cell wall cross-linking, a reaction catalyzed by Class III peroxidases under high H_2_O_2_ conditions [27], thereby restricting root elongation [29]. By completely repressing *TaPOD1*, Sarajevo 1 may avoid this maladaptive cell wall rigidification, maintaining architectural plasticity while relying on basal metabolic quiescence. Class III peroxidases constitute a large, plant-specific multigene family whose members play well-established roles in ROS homeostasis under salinity [64,65]; however, their unregulated maintenance in CI 17260 may represent a metabolic burden rather than an effective defense strategy when global energy conservation is the adaptive imperative. This transcriptional pattern is qualitatively consistent with the H_2_DCFDA fluorescence imaging results (Section 3.6; Supplementary Figure S5), which showed substantially attenuated fluorescence signals in root tips of all three genotypes under salt stress relative to control conditions. However, since the H_2_DCFDA assay was performed qualitatively in this study, a direct causal link between *TaPOD1*/*TaPOD-2D* transcriptional repression and the observed fluorescence attenuation cannot be established from the present data.

The failure of CI 17260 to completely silence *TaPOD1* is consistent with a broader pattern of deficient transcriptional control, as ROS-mediated suppression of PIN-FORMED expression and accelerated peroxidase-catalyzed oxidative IAA catabolism would further compromise auxin homeostasis at meristematic zones [66]. Similarly, *TaPIN-like* (TraesCSU02G083100, log_2_FC = 6.20 in CI 17260 vs. Sarajevo 1, FDR = 1.08 × 10^−17^) encodes a putative auxin efflux carrier that is near-zero in Sarajevo 1 but aberrantly induced in CI 17260 under salt stress. Salinity typically downregulates PIN1, PIN3, and PIN7 expression through nitric oxide- and ROS-mediated signaling, reducing basipetal and lateral auxin transport capacity and causing root meristem size reduction [22,23]. The aberrant induction of *TaPIN-like* specifically in CI 17260—against this canonical suppressive background—suggests a potentially dysregulated response, consistent with a pattern of auxin redistribution that may not be effectively coordinated under conditions of compromised ROS homeostasis. In contrast, the strict repression of *TaPIN-like* in Sarajevo 1 reflects the energetic logic of metabolic quiescence: redundant auxin redistribution pathways are shut down to conserve resources during the stress response. Although functional validation of *TaPIN-like* efflux activity in wheat roots under salt stress remains to be demonstrated experimentally, its differential regulation between contrasting genotypes identified in this study provides a transcriptomic basis for future transgenic or CRISPR-based validation in wheat.

*TaExpansin* (TraesCS1D02G299700; log_2_FC = 10.71 in CI 17260 vs. Sarajevo 1, FDR = 3.34 × 10^−92^) encodes a cell wall-loosening factor that was expressed at near-zero levels in Sarajevo 1 but aberrantly induced from basal levels in CI 17260 under salt stress. Expansins mediate cell wall loosening by disrupting non-covalent hydrogen bonds within the polysaccharide matrix of the primary cell wall, reducing tensile stress and enabling turgor-driven cell elongation [29,30]. In the context of high salinity, where elevated extracellular Na^+^ imposes severe osmotic constraints on turgor pressure [67], the induction of *TaExpansin* in CI 17260 under stress—in the absence of adequate auxin delivery and ROS control—likely represents an inefficient investment in cell wall expansion under these osmotic conditions. Furthermore, the concurrent incomplete repression of TaPOD1 in CI 17260 may exacerbate cell wall rigidification through peroxidase-mediated oxidative cross-linking of cell wall polysaccharides, under conditions of incomplete ROS attenuation [27]. This dual failure—aberrant expansin induction and concurrent peroxidase-mediated wall stiffening—represents a mechanistically dysregulated and energetically costly cell wall response, consistent with the severe architectural deterioration observed in CI 17260 (Table 1).

Collectively, the pattern of aberrant induction and incomplete repression across these three modules in CI 17260 (*TaExpansin* > *TaPIN-like* >> *TaPOD1*) is proposed to reflect dysregulated transcriptional control collectively associated with severe root architectural deterioration (Table 1). In direct contrast, the complete and coordinated transcriptional repression of these same modules in Sarajevo 1 suggests that the capacity for precise transcriptional shutdown—rather than active stress-induced defense—may be a central feature of profound metabolic quiescence and the key factor underlying superior salt tolerance. Although the transcriptional patterns suggest differential metabolic strategies between genotypes, direct measurements of energy expenditure were not performed. Therefore, the interpretation of metabolic efficiency or inefficiency should be considered as an inference based on transcriptomic data.

### 4.4. Strategic Implications for Salt-Resilient Wheat Breeding

The integration of our genetic, transcriptomic, and phenotypic findings provides a mechanistic framework with potential translational value for wheat breeding programs targeting saline environments. Our identification of the *TaIAO* locus on chromosome 7B as a constitutive determinant of root architectural complexity offers a promising entry point for marker-assisted selection (MAS). Notably, the favorable *CC* haplotype at SNP AX-95079518, which occurs at a high frequency of 67.7% in our core collection, is associated with superior baseline root architecture and enhanced stress-responsive transcriptional control. This favorable major allele may serve as a reliable diagnostic marker for pre-screening germplasm collections for salt tolerance potential. Marker-assisted selection based on constitutive genetic factors offers particular advantages for breeding programs, as it may enable phenotype prediction without requiring stress exposure [68]. Unlike adaptive traits that manifest only under severe stress conditions, the constitutive nature of this locus allows breeders to evaluate candidate genotypes under non-stress environments, reducing the logistical and economic costs associated with large-scale phenotyping under saline conditions [69].

Beyond single-locus selection, our findings advocate for a systems-oriented breeding strategy that prioritizes the integration of constitutive and inducible components. Specifically, pyramiding favorable alleles at the chromosome 7B auxin biosynthesis locus with precision transcriptional repression capacity—as exemplified by the complete shutdown of *TaPOD1*, *TaPIN-like*, and *TaExpansin* in Sarajevo 1—would simultaneously maintain basal developmental vigor and stress-responsive metabolic quiescence. This two-tier genetic architecture may partially explain why conventional selection for salt tolerance—which often focuses exclusively on stress-induced responses—has faced significant challenges [70]. Future breeding strategies should integrate marker-assisted selection for favorable *TaIAO* haplotypes with the introgression of alleles associated with differential regulation of *TaPIN-like* and *TaPOD1* to simultaneously maintain basal developmental vigor and stress-responsive metabolic quiescence. Furthermore, transgenic or genome-editing approaches targeting the regulatory elements of these stress-responsive genes may offer additional avenues for engineering salinity tolerance in elite wheat cultivars while maintaining yield performance under non-stress conditions. As climate-driven soil salinization continues to threaten global wheat production, breeding programs that incorporate this mechanistic, multi-tier strategy may provide effective strategies for developing cultivars capable of maintaining yield stability in marginal and salt-affected lands.

## 5. Conclusions

By integrating genome-wide association mapping with comparative transcriptomic profiling, this study provides evidence that wheat root plasticity under salt stress is governed by a coordinated two-tier genetic architecture that integrates constitutive genetic determinants with stress-responsive transcriptional repression, rather than relying solely on energetically costly active defense responses.

At the first tier, the *TaIAO* locus on chromosome 7B (*TraesCS7B02G418900*; favorable CC haplotype at SNP AX-95079518) was associated with superior baseline root architectural complexity, consistent with a candidate role in constitutive auxin biosynthetic capacity. This association was reflected in the markedly greater pre-stress root development observed in Sarajevo 1 relative to the sensitive genotype CI 17260. This pre-established root system may provide a developmental foundation for more effective stress buffering under severe osmotic challenge. The proposed mechanistic link between this locus and auxin biosynthetic capacity is supported by ELISA-based IAA quantification (Section 3.5; Table 6), although direct biochemical characterization of *TaIAO* substrate specificity remains to be performed.

At the second tier, the defining characteristic of salt tolerance in Sarajevo 1 was the capacity for complete and coordinated transcriptional repression of three energy-consuming gene modules under stress: cell wall expansion (*TaExpansin*), auxin redistribution (*TaPIN-like*), and constitutive ROS defense (*TaPOD1*). This pattern of precision repression was consistent with pronounced metabolic quiescence and coincided with the preservation of 95.2% of root length and an increase in root volume to 113.3% of control values. In contrast, CI 17260 aberrantly induced or failed to completely repress these same modules, a pattern associated with severe root architectural deterioration (61.1% root length retention; Table 1).

Together, these findings suggest a mechanistic framework in which wheat salt tolerance may depend not only on stress-induced defense responses but also on constitutive root developmental capacity coupled with precision transcriptional repression of energy-consuming modules under stress. This framework may offer a basis for developing marker-assisted selection (MAS) strategies targeting both the *TaIAO* locus and the differential regulation of auxin–ROS effector modules. Direct enzymatic characterization of *TaIAO* substrate specificity (EC 1.2.3.7), in planta functional validation, and studies across diverse genetic backgrounds will be necessary to substantiate and extend the proposed regulatory model.

## Figures and Tables

**Table 1:** Phenotypic variation in root traits among wheat genotypes under control and salt stress conditions.

Trait	Genotype	Control	Salt Treated (200 mM NaCl)	Relative Value (% of Control)
Total Root Length (cm)	Keumkang (Standard)	116.7 ± 8.5	64.5 ± 2.4	55.3 ± 4.3 ^NS^
	CI 17260 (Sensitive)	90.2 ± 4.5	55.1 ± 1.6	61.1 ± 4.7 ^NS^
	Sarajevo 1 (Tolerant)	158.7 ± 8.8	151.0 ± 7.0	95.2 ± 5.1 ^NS^
Surface Area (cm^2^)	Keumkang	12.8 ± 0.7	8.1 ± 0.3	63.0 ± 3.8 ^NS^
	CI 17260	10.0 ± 0.5	6.8 ± 0.2	68.2 ± 4.1 ^NS^
	Sarajevo 1	15.5 ± 0.8	16.0 ± 0.6	103.6 ± 5.4 ^NS^
Root Volume (mm^3^)	Keumkang	112.0 ± 7.0	81.0 ± 2.0	72.1 ± 4.9 ^b^
	CI 17260	89.0 ± 4.0	68.0 ± 2.0	76.4 ± 4.9 ^b^
	Sarajevo 1	120.0 ± 5.0	136.0 ± 5.0	113.3 ± 5.5 ^a^
Number of Root Tips (count)	Keumkang	153.2 ± 16.5	111.3 ± 8.2	72.6 ± 6.1 ^NS^
	CI 17260	93.3 ± 7.9	64.9 ± 5.6	69.6 ± 5.8 ^NS^
	Sarajevo 1	277.3 ± 27.6	265.8 ± 17.1	95.8 ± 6.5 ^NS^

Values are presented as the mean ± SE (*n* = 12; three independent biological replicates with four individual plants per replicate). Relative Value indicates the percentage of salt-stressed value relative to control (Stress/Control × 100). Genotypic differences in relative values were evaluated by one-way ANOVA followed by Tukey’s HSD post hoc test. Different superscript letters within the Relative Value column indicate statistically significant differences among genotypes (*p* < 0.05); NS, not significant (*p* > 0.05). Root volume (RV) was the only trait showing significant genotypic differences in salt-stress retention capacity (Sarajevo 1 > Keumkang = CI 17260; *p* < 0.05). Average root diameter (ARD) was measured and used for GWAS but is excluded from this table, as no significant genotypic difference in relative values was detected (*p* > 0.05).

**Table 2:** Summary of significant marker-trait associations (MTAs) and candidate genes for root architectural traits identified by GWAS under salt stress.

Trait	Chr.	SNP ID	Position (bp) ^1^	FarmCPU −*log*_10_(*p*)	MLM *p*-Value	Candidate Gene ID	Putative Function
Root volume	7B	AX-95079518	687,324,247	4.5	4.49 × 10^−5^	*TraesCS7B02G418900*	Indole-3-acetaldehyde oxidase (Auxin biosynthesis)
	7B	AX-95181207	687,659,784	≥4.40	5.65 × 10^−5^	(Flanking marker)	-
Root diameter	6D	AX-94853273	21,702,992	4.6	5.36 × 10^−5^	*TraesCS6D02G047000*	F-box (Ubiquitin-mediated degradation)

^1^ Position: Physical position based on the wheat reference genome (IWGSC RefSeq v1.0). The primary GWAS analysis was performed using the FarmCPU model with normalized trait values (NaCl/Control ratio); listed −log_10_(*p*) values correspond to FarmCPU results. The Mixed Linear Model (MLM) *p*-value is provided as an independent cross-validation to assess consistency across models. All listed SNPs exceeded the suggestive threshold of −log_10_(*p*) ≥ 4.36 under the FarmCPU model. Root Volume: The candidate gene *TraesCS7B02G418900* is physically located between the two significant SNPs (AX-95079518 and AX-95181207), defining a QTL interval of approximately 335 kb.

**Table 3:** Summary of differentially expressed genes (DEGs) in wheat root transcriptomes under salt stress.

Comparison	Genotype Condition	Total DEGs	Up- Regulated	Down- Regulated	Transcriptomic Strategy
Comp 1	Sarajevo 1 (NaCl vs. Con)	18,416	7883	10,533	Coordinated transcriptional repression (Downregulation dominant)
Comp 2	CI 17260 (NaCl vs. Con)	16,155	8246	7909	Balanced response
Comp 3	Keumkang (NaCl vs. Con)	17,349	9069	8280	Transcriptional induction (Upregulation dominant)
Comp 4	Sarajevo 1 vs CI 17260 (Under NaCl)	13,707	8131	5576	Incomplete repression (Dysregulated induction in salt- sensitive CI 17260)

DEGs were identified based on a false discovery rate (FDR < 0.05) and |log_2_FC| ≥ 1. Comparisons 1–3 show the transcriptomic response to salt stress (NaCl vs. Control) within each genotype: Sarajevo 1 exhibited a predominant downregulation pattern (10,533 downregulated genes), consistent with a metabolic quiescence response; Keumkang showed the highest number of upregulated genes (9069), consistent with an active transcriptional response; CI 17260 displayed a relatively balanced response (8246 upregulated vs. 7909 downregulated genes). Comparison 4 directly contrasts the tolerant (Sarajevo 1) and salt-sensitive (CI 17260) genotypes under salt stress, revealing a marked divergence: predominant downregulation in Sarajevo 1 versus aberrant induction or incomplete repression of specific gene modules in CI 17260.

**Table 4:** Differentially expressed genes showing aberrant induction or incomplete transcriptional repression in the salt-sensitive genotype CI 17260 compared with the tolerant genotype Sarajevo 1 under salt stress.

Functional Category	Gene Name	Gene ID	Log_2_FC ^a^	FDR	Expression Pattern (Under NaCl)
Cell wall Remodeling	*TaExpansin ^c^*	*TraesCS1D02G299700*	10.71	3.34 × 10^−92^	Aberrantly induced from basal levels in CI 17260; strictly repressed in Sarajevo 1
ROS Scavenging	*TaPOD1*	*TraesCS7D02G417400*	10.51	6.95 × 10^−81^	Incompletely repressed in CI 17260 (~199 reads); completely shut down in Sarajevo 1 (read counts = 0)
Auxin Transport	*TaPIN-like ^c^*	*TraesCSU02G083100*	6.20	1.08 × 10^−17^	Aberrantly induced from basal levels in CI 17260; strictly silenced in Sarajevo 1
Auxin Biosynthesis	*TaIAO ^b^*	*TraesCS7B02G418900*	N/A	N/A	Constitutive expression; not differentially expressed under stress in either genotype (below DEG threshold), consistent with a pre-stress developmental role

^a^ log_2_FC: Log_2_ Fold Change values represent the expression ratio of the salt-sensitive CI 17260 (Ta_1694) relative to the salt-tolerant Sarajevo 1 (Ta_1936) under salt stress conditions (Comparison 4); positive values indicate higher expression in CI 17260. All listed genes met the significance criteria of FDR < 0.05 and |log_2_FC| ≥ 1, except *TaIAO* (see footnote ^b^). ^b^ *TaIAO* (indole-3-acetaldehyde oxidase-like) was identified as a major locus via GWAS (SNP: AX-95079518). Transcript levels of this gene were below the statistical threshold for DEGs under salt stress, consistent with a role in constitutive root architectural development rather than stress-induced transcriptional activation. ^c^ In CI 17260, *TaExpansin* and *TaPIN-like* were identified as stress-induced genes (NaCl vs. Control): *TaExpansin* log_2_FC = 4.73, FDR = 2.65 × 10^−91^; *TaPIN-like* log_2_FC = 2.90, FDR = 9.30 × 10^−13^. These data indicate that the elevated expression observed between the two genotypes under stress reflects aberrant stress induction in CI 17260 rather than a constitutive genotypic difference. *TaPIN-like* was not detected in Sarajevo 1 under either condition, consistent with complete transcriptional repression in the tolerant genotype. This table presents a mechanistically focused subset of DEGs directly relevant to the Two-Tier Adaptive Strategy proposed in this study; it is not intended as an exhaustive representation of all DEGs identified. The complete DEG dataset is provided in Supplementary Figure S4.

**Table 5:** Key candidate genes showing divergent expression between the salt-tolerant genotype (Sarajevo 1) and the salt-sensitive genotype (CI 17260) under salt stress.

Identification Method	Candidate Gene	Gene ID	Chr.	log_2_FC ^a^	FDR ^a^	Functional Annotation
GWAS	*TaIAO*	*TraesCS7B02G418900*	7B	N/A ^b^	N/A ^b^	Indole-3-acetaldehyde oxidase (Auxin biosynthesis)
RNA-Seq	*TaExpansin*	*TraesCS1D02G299700*	1D	10.71	3.34 × 10^−92^	Expansin (Cell wall remodeling)
RNA-Seq	*TaPOD1*	*TraesCS7D02G417400*	7D	10.51	6.95 × 10^−81^	Class III peroxidase (ROS scavenging)
RNA-Seq	*TaPIN-like*	*TraesCSU02G083100*	Un ^c^	6.2	1.08 × 10^−17^	Auxin efflux carrier (Auxin transport)

^a^ log_2_FC and FDR: Values represent the expression ratio of the salt-sensitive CI 17260 (Ta_1694) relative to the salt-tolerant Sarajevo 1 (Ta_1936) under salt stress conditions (Comparison 4); positive values indicate higher expression in CI 17260. All listed RNA-seq candidate genes met the significance criteria of FDR < 0.05 and |log_2_FC| ≥ 1. ^b^ *TaIAO* was identified as a candidate locus associated with root volume via GWAS (SNP: AX-95079518). N/A indicates that transcript levels of this gene were below the statistical threshold for DEGs under salt stress, consistent with a role in constitutive root architectural development prior to stress, rather than as a component of stress-induced transcriptional activation. ^c^ *TraesCSU02G083100* is located on an unassigned scaffold (Un) in the IWGSC RefSeq v1.0 assembly; its functional annotation and differential expression pattern are nonetheless well-supported by the transcriptomic data.

**Table 6:** Endogenous IAA content in root tissues of three wheat genotypes under control and salt stress conditions.

Genotype	Control (ng/g FW)	200 mM NaCl (ng/g FW)	Change (%)	*p*-Value ^c^
Sarajevo 1	112.8 ± 5.0 ^a^	91.1 ± 3.9 ^b^	−19.2	0.030 *
Keumkang	109.2 ± 5.0 ^a^	103.8 ± 0.9 ^a^	−5.0	0.392 ns
CI 17260	93.7 ± 2.4 ^b^	104.5 ± 2.6 ^a^	+11.6	0.037 *

Values are presented as mean ± SE (*n* = 3). Lowercase superscript letters within each column indicate Duncan’s multiple range test (MRT) groupings for between-genotype comparisons within each treatment condition. Different letters denote statistically significant differences at *p* < 0.05 (one-way ANOVA: Control, *F*(2,6) = 5.52, *p* = 0.044; 200 mM NaCl, *F*(2,6) = 7.45, *p* = 0.024). ^c^ *p*-values are from Welch’s *t*-test for within-genotype comparison between control and salt treatment conditions. *, *p* < 0.05; ns, not significant.

## Data Availability

The datasets presented in this study can be found in online repositories. The names of the repository/repositories and accession number can be found below: https://kbds.re.kr/ (accessed on 12 June 2026), accession number KAP230670.

## Supplementary Materials

The following supporting information can be downloaded at: https://www.mdpi.com/article/10.3390/biology15120965/s1, Figure S1: Hydroponic culture system used for wheat seedling growth and salt stress treatment.; Figure S2: Representative photographs of wheat seedlings under control and salt stress conditions; Figure S3: Genome-wide association study (GWAS) results using the Mixed Linear Model (MLM) for Root Volume under salt stress.; Figure S4: Global transcriptomic changes in wheat roots under salt stress.; Figure S5: H_2_DCFDA fluorescence imaging of reactive oxygen species (ROS) accumulation in root tips of three wheat genotypes under control and 200 mM NaCl conditions; Figure S6: Quantitative real-time PCR (qRT-PCR) analysis of nine candidate gene homeologs in roots of three wheat genotypes under control and salt stress (200 mM NaCl, 5 days) conditions; Table S1: Top significant SNPs identified by Mixed Linear Model (MLM) analysis for root morphological traits under salt stress; Table S2. Primer sequences used for quantitative real-time PCR (qRT-PCR) analysis of candidate genes in this study; Table S3. Chromosomal correspondence between qRT-PCR target loci and RNA-seq-identified differentially expressed genes (DEGs) for candidate genes analyzed in this study.
