# Supplementary material for: Integrated Genomic and Transcriptomic Analyses Reveal a Two-Tier Adaptive Strategy for Wheat Root Salt Tolerance: Constitutive Auxin Biosynthetic Capacity and Stress-Responsive Transcriptional Repression
Source: Biology (Basel). 2026 Jun 19;15(12):965. doi: 10.3390/biology15120965 (PMC13295696; doi:10.3390/biology15120965)
Supplement: Supplementary file 1 [file biology-15-00965-s001.zip › Supplementary_Figure S1-S6.pdf]

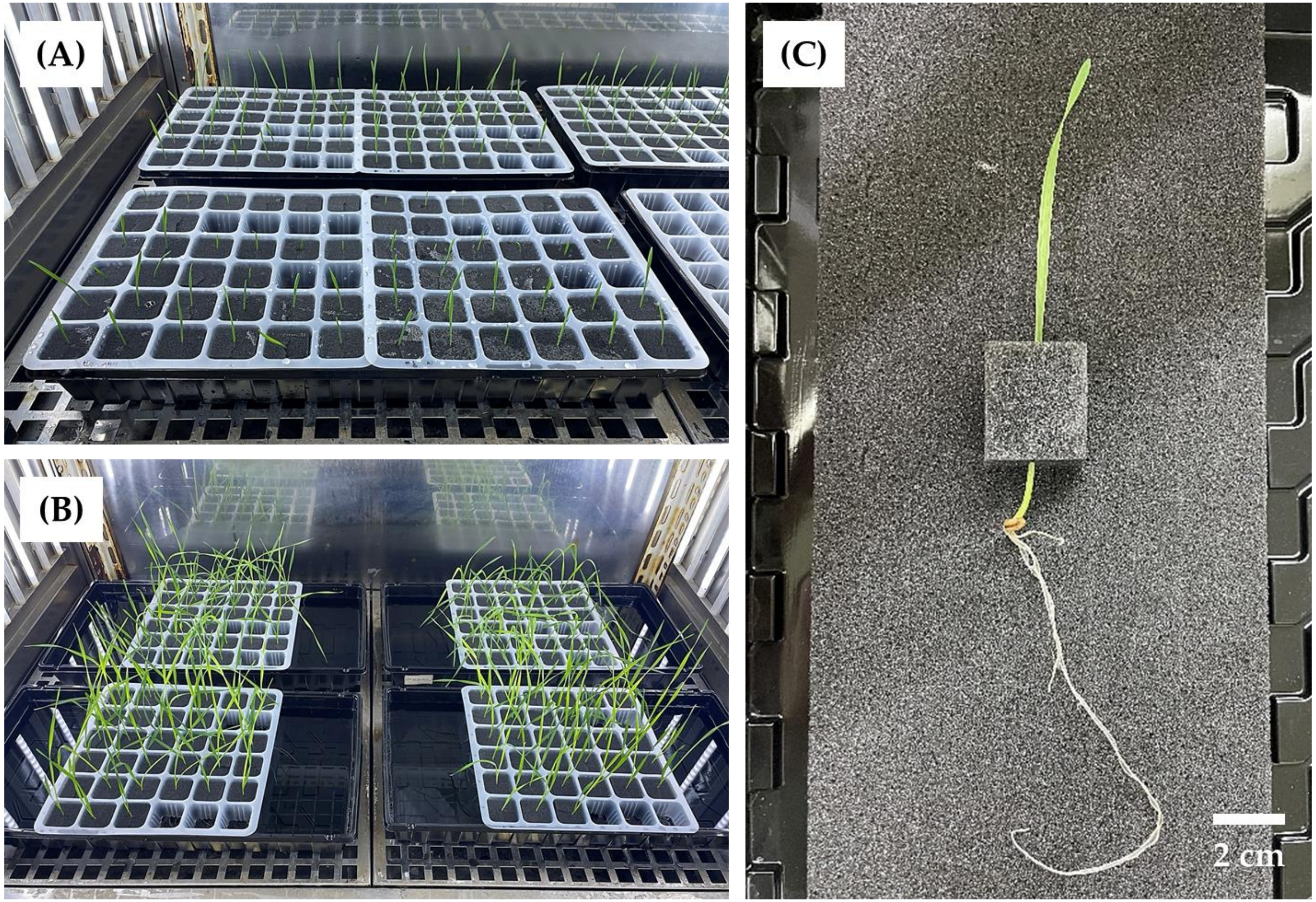

Figure S1. Hydroponic culture system used for wheat seedling growth and salt stress treatment. (A) Top-angle overview of 36-cell plug trays containing uniformly germinated wheat (*Triticum aestivum*) seedlings at the early growth stage (approximately 7 days after sowing), arranged in a controlled growth chamber. (B) Overview of the hydroponic tray setup at the transplanting stage (approximately 10 days after sowing) prior to NaCl treatment, showing 36-cell sponge-supported seedlings placed in black water reservoir trays within a controlled growth chamber (16 h light/8 h dark; 23 °C/18 °C day/night; 60% relative humidity). (C) Representative individual wheat seedling supported by a sponge insert (~35 mm), illustrating the shoot and seminal root system morphology at the time of salt treatment initiation. Scale bar = 2 cm.

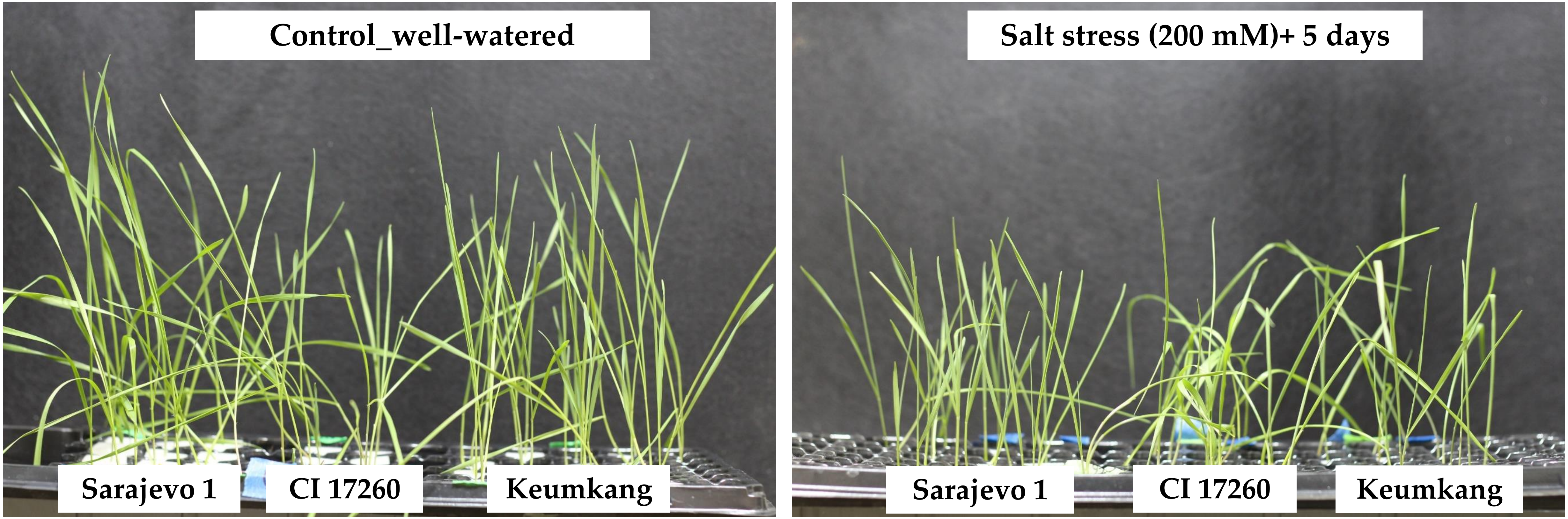

Figure S2. Representative photographs of wheat seedlings grown under control conditions (left panel) or exposed to 200 mM NaCl for five days (right panel), taken at the time of root tissue sampling. Genotypes are arranged from left to right: Sarajevo 1 (salt-tolerant), CI 17260 (salt-sensitive), and Keumkang (standard cultivar). Salt treatment resulted in apparent reductions in shoot growth in all three genotypes relative to control conditions.

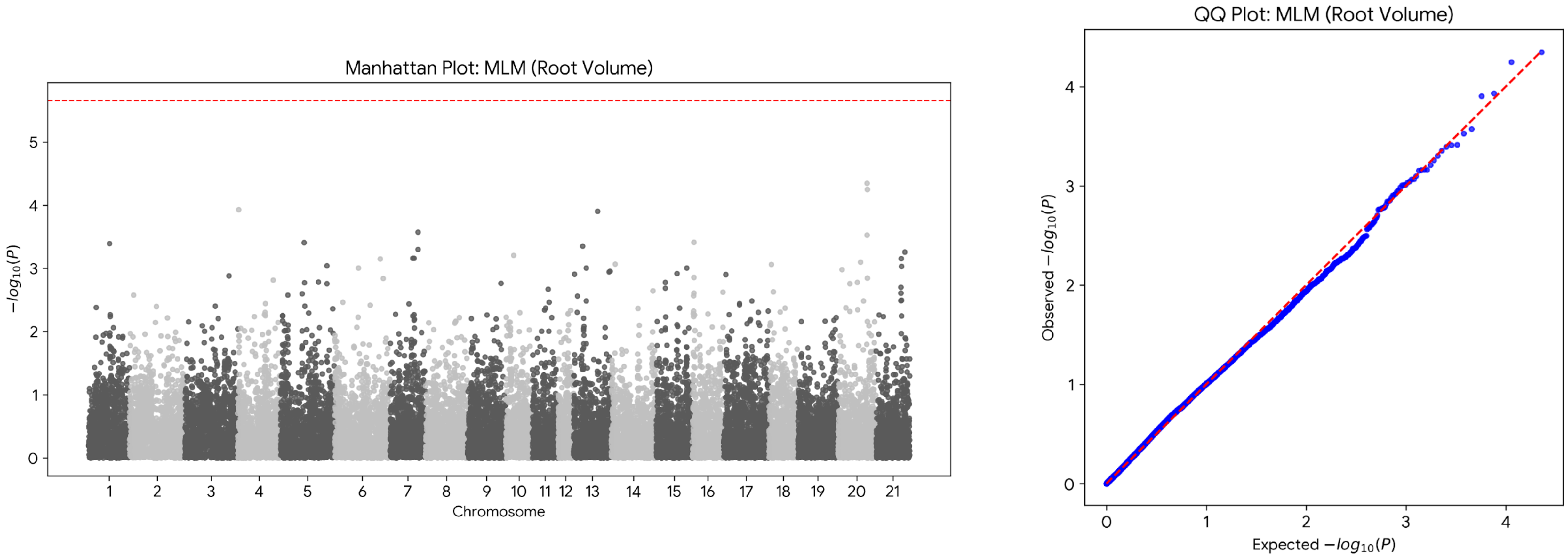

(A) Manhattan Plot for Root Volume

(B) QQ Plot

Figure S3. Genome-wide association study (GWAS) results using the Mixed Linear Model (MLM) for root volume under salt stress. (A) Manhattan plot displaying the association signals across the wheat chromosomes. The red dashed line represents the genome-wide significance threshold. (B) Quantile-Quantile (QQ) plot showing the expected versus observed $-\log_{10}(p)$ values. The distribution of observed *p*-values is consistent with adequate control of false positives, supporting the primary association signals detected by the FarmCPU model.

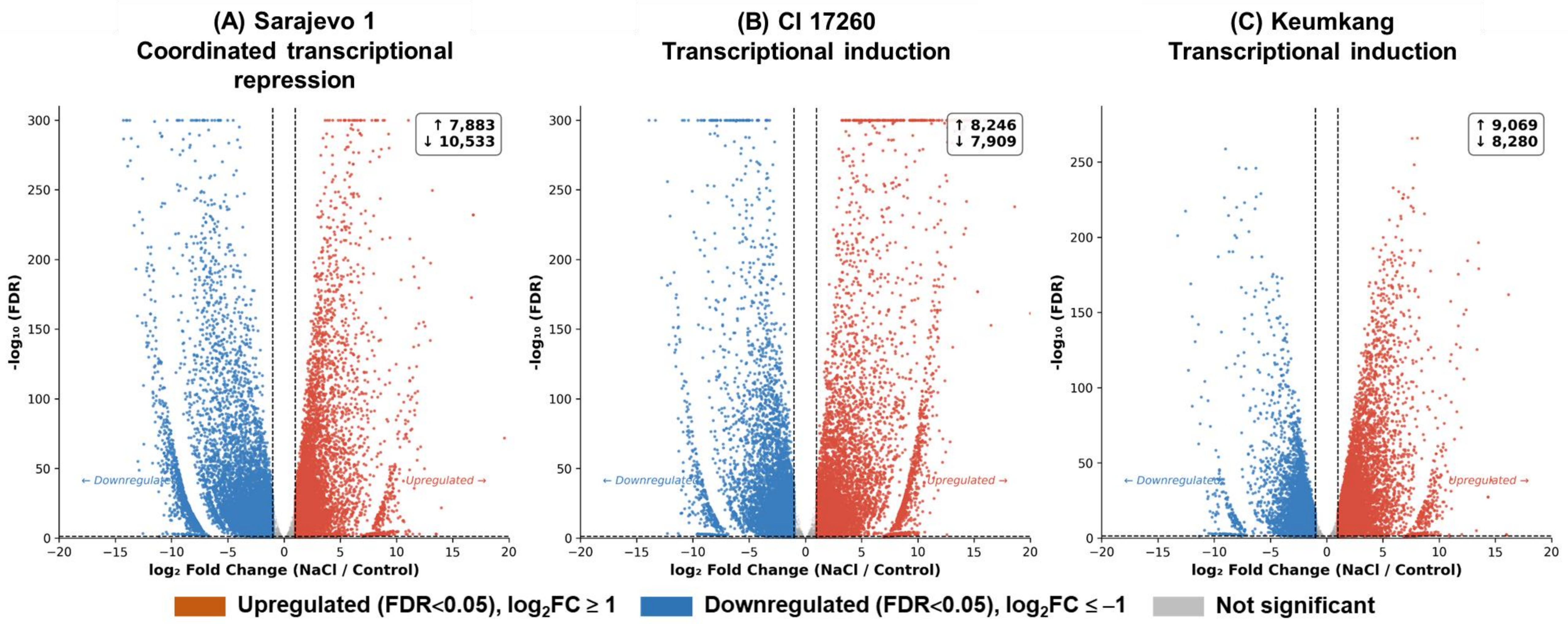

Figure S4. Global transcriptomic changes in wheat roots under salt stress. Volcano plots illustrating the distribution of DEGs for (A) Sarajevo 1, (B) CI 17260, and (C) Keumkang. The x-axis represents $\log_2$ fold change (NaCl/Control), and the y-axis represents the significance level ($-\log_{10}$ FDR). Red and blue dots indicate significantly upregulated and downregulated genes, respectively (FDR < 0.05, $|\log_2 FC| \geq 1$), while grey dots represent non-significant genes. Note the higher density of upregulated genes (right side) in Keumgang (C), contrasting with the pronounced shift toward downregulation (left side) in Sarajevo 1 (A; 10,533 downregulated vs. 7,883 upregulated DEGs), a pattern consistent with broad transcriptional repression under salt stress.

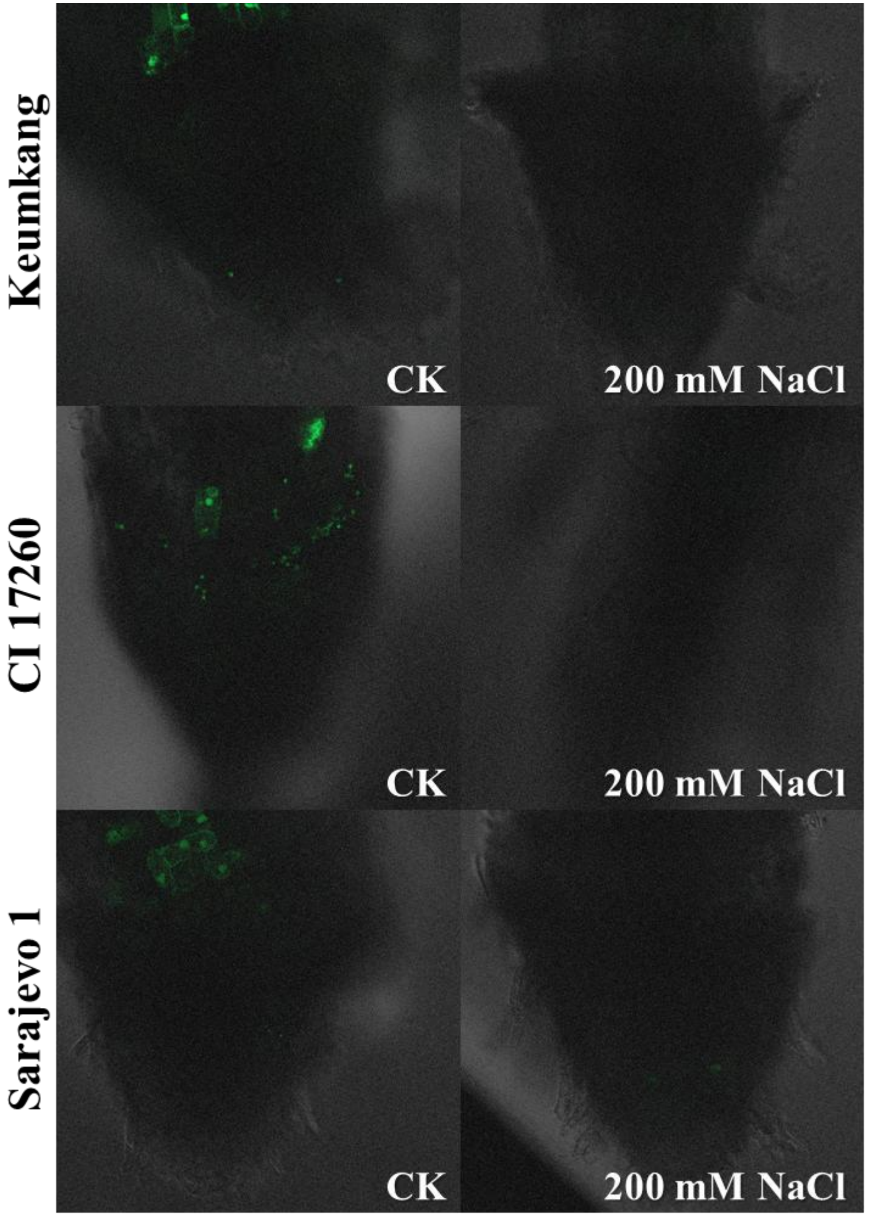

Figure S5. H_2DCFDA fluorescence imaging of reactive oxygen species (ROS) accumulation in root tips of three wheat genotypes (Keumkang, CI 17260, and Sarajevo 1) under control and 200 mM NaCl conditions. Green fluorescence indicates intracellular ROS. Following five-day salt exposure, fluorescence was substantially reduced in root tips across all three genotypes relative to controls.

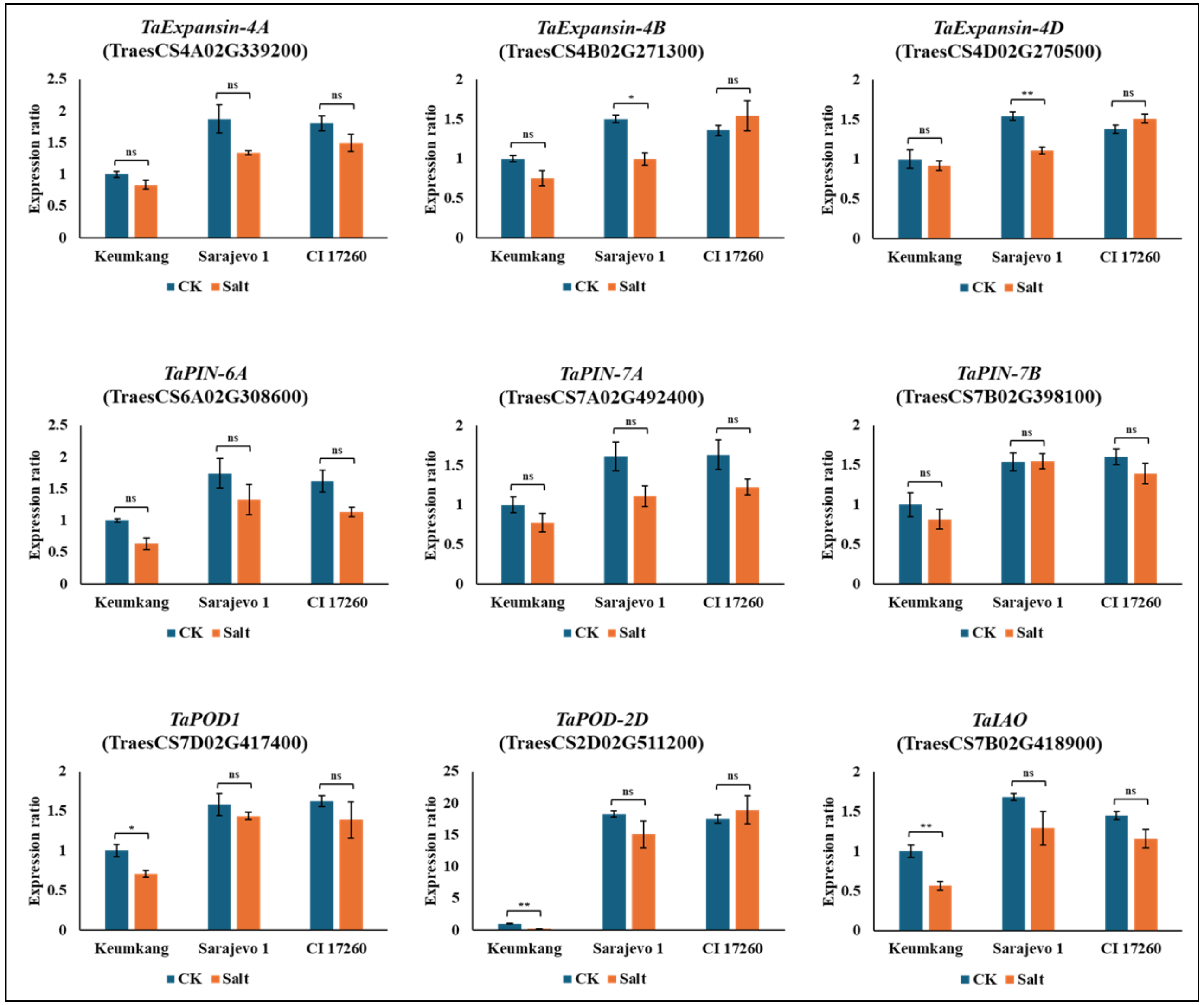

Figure S6. Quantitative real-time PCR (qRT-PCR) analysis of nine candidate gene homeologs in roots of three wheat genotypes (Keumkang, Sarajevo 1, and CI 17260) under control (CK) and salt stress (200 mM NaCl, 5 days) conditions. Relative expression levels of (A) *TaExpansin-4A* (TraesCS4A02G339200), *TaExpansin-4B* (TraesCS4B02G271300), and *TaExpansin-4D* (TraesCS4D02G270500); (B) *TaPIN-6A* (TraesCS6A02G308600), *TaPIN-7A* (TraesCS7A02G492400), *TaPIN-7B* (TraesCS7B02G398100), *TaPOD1* (TraesCS7D02G417400), and *TaPOD-2D* (TraesCS2D02G511200); and (C) *TaIAO* (TraesCS7B02G418900) were determined by qRT-PCR using TaActin (AF326781) as the internal reference gene. Seedlings of Keumkang, Sarajevo 1, and CI 17260 were treated with 200 mM NaCl (Salt) or tap water (CK) for 5 days. Expression ratios are presented as mean ± SE (n = 3 biological replicates). Statistical significance of within-genotype CK-to-salt comparisons was assessed by one-way ANOVA with post hoc comparison or Welch's t-test, as appropriate. ns, not significant ($p > 0.05$); *, $p < 0.05$; **, $p < 0.01$.
