# Supplementary material for: Integrated Genomic and Transcriptomic Analyses Reveal a Two-Tier Adaptive Strategy for Wheat Root Salt Tolerance: Constitutive Auxin Biosynthetic Capacity and Stress-Responsive Transcriptional Repression
Source: Biology (Basel). 2026 Jun 19;15(12):965. doi: 10.3390/biology15120965 (PMC13295696; doi:10.3390/biology15120965)
Supplement: Supplementary file 1 [file biology-15-00965-s001.zip › Supplementary_Table S1.pdf]

Table S1. Top significant SNPs identified by Mixed Linear Model (MLM) analysis for root morphological traits under salt stress.

Root traits	SNP	Chr	Pos	*P*. value	MAF	Effect
Root volume	AX-95079518	7B	687,324,247	4.49E-05	0.32	-0.077
	AX-95181207	7B	687,659,784	5.56E-05	0.44	-0.084
	AX-94650880	2B*	8,719,549	1.17E-04	0.08	0.115
Root Diameter	AX-95178106	2D*	519,133,490	1.48E-05	0.13	0.124
	AX-94738362	2B*	790,752,072	2.95E-05	0.14	0.178
	AX-94853273	6D	21,702,992	5.36E-05	0.06	0.146

The table lists the most significant single-nucleotide polymorphisms (SNPs) associated with Root Volume and Root Diameter. **Chr**, chromosome; **Pos**, physical position on the chromosome in base pairs (bp) based on the IWGSC RefSeq v1.0; **MAF**, minor allele frequency; **Effect**, allelic substitution effect of the minor allele on the trait. Asterisks (*) indicate estimated chromosome assignments based on the reference genome.
